# The conserved single-cleavage mechanism of animal DROSHA enzymes

**DOI:** 10.1038/s42003-021-02860-1

**Published:** 2021-11-25

**Authors:** Thuy Linh Nguyen, Trung Duc Nguyen, Tuan Anh Nguyen

**Affiliations:** Division of Life Science, The Hong Kong University of Science & Technology, Hong Kong, China

**Keywords:** Enzyme mechanisms, miRNAs

## Abstract

RNase III enzymes typically cleave both strands of double-stranded RNAs (dsRNAs). We recently discovered that a human RNase III, DROSHA, exhibits a single cleavage on the one strand of primary microRNAs (pri-miRNAs). This study revealed that DROSHAs from the other animals, including worms and flies, also show the single cleavage on dsRNAs. Furthermore, we demonstrated that the mechanism of single cleavage is conserved in animal DROSHA enzymes. In addition, the dsRNA-binding domain (dsRBD) and a 3p-strand cleavage-supporting helix (3pCSH) of the DROSHA enzymes foster a weak single cleavage on one strand, which ensures their double cleavages. Disrupting the interaction of dsRBD-RNA and 3pCSH-RNA by an internal loop (IL) and a 3pCSH-loop in the lower stem of pri-miRNAs, respectively, inhibits one of the double cleavages of DROSHAs, and this results in the single cleavage. Our findings expand our understanding of the enzymatic mechanisms of animal DROSHAs. They also indicate that there are currently unknown cellular functions of DROSHA enzymes using their single cleavage activity.

## Abstract (web summary)

Recently, the human RNase III enzyme DROSHA had been shown to exhibit single cleavage activity. Nguyen et al. report that this molecular mechanism of DROSHA is conserved in worms and flies, suggesting a common feature of animal DROSHAs.

## Introduction

Prokaryotic and eukaryotic RNase IIIs are a large group of enzymes that make double cleavages on RNA duplexes and play essential roles in various RNA metabolic pathways^1,2^. Only two RNase III enzymes, DROSHA and DICER, have been identified in humans, and both are critical for producing microRNAs (miRNAs). miRNAs are a class of small non-coding RNAs that post-transcriptionally regulate gene expression^3^. miRNAs are widespread throughout the animal kingdom, from worms to humans^4^. Animal cells synthesize most miRNAs using a conserved canonical miRNA biogenesis pathway. In this pathway, miRNAs are excised from longer primary miRNA transcripts (pri-miRNAs) by two cleavage steps. In the nucleus, the Microprocessor complex (MP), which is composed of DROSHA and the double-stranded RNA (dsRNA)-binding protein, DGCR8, executes the first double-cleavage in the dsRNA stem of pri-miRNAs to generate hairpins (pre-miRNAs) of ~60 nt long. Exportin-5 then transports the pre-miRNAs to the cytoplasm, where DICER makes the second double-cleavage in the pre-miRNAs to generate ~22-bp miRNA duplexes^3,5^. The miRNA duplexes are then loaded onto a protein called Argonaute (AGO). One strand is discarded while the other serves as the guide RNA for targeting mRNAs, leading to silent gene expression^6,7^. Efficient double cleavages by DROSHA and DICER are crucial for controlling an adequate expression of miRNA in humans^5,7^.

**Fig. 1:**
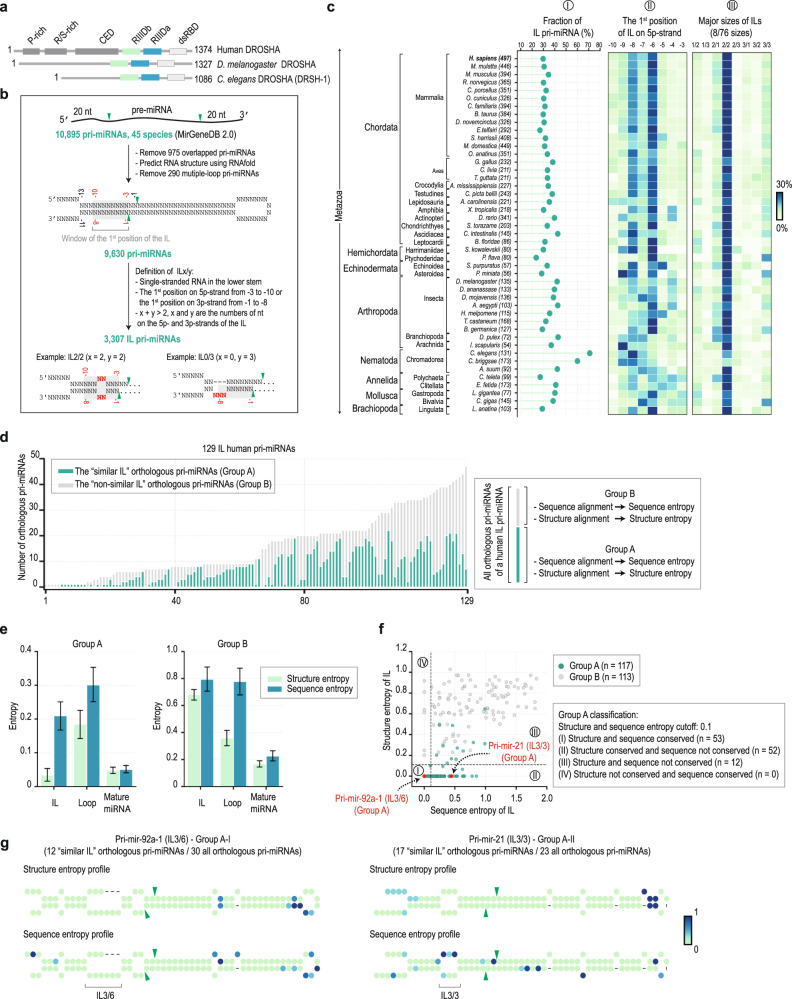
The internal loop (IL) in the lower stem is a common feature of animal pri-miRNAs. **a** The domain composition of DROSHA in *H. sapiens* (humans), *D. melanogaster*, and *C. elegans*. P-rich, proline-rich; RS, arginine-serine-rich; CED, central domain; RIIIDa and RIIIDb, RNase III domains; and dsRBD, double-stranded RNA-binding domain. **b** The 10,895 pri-miRNA sequences were collected from MirGeneDB 2.0. The secondary structures of these pri-miRNAs were predicted using RNAfold. The IL was defined as the internal loop containing more than 3 nt in the lower stem of pri-miRNAs. The green arrowheads represent the cleavage sites of DROSHA. **c** The percentage of IL-pri-miRNAs in 45 species (panel I). The ILs are usually located at 6 and 8 nt from the cleavage sites of DROSHA on the 5p-strand of pri-miRNAs (panel II). The major sizes of ILs are 2/2 (panel III). There are a total of 76 sizes of ILs found in all 45 species. The 8 main sizes of ILs were shown. The fraction values were colored according to the color bar (right). **d** The number of the similar IL-pri-miRNA orthologs (group A) and non-similar IL-pri-miRNA orthologs (group B) for each of 129 human IL-pri-miRNAs. **e** The average of sequence and structure entropies was estimated for the ILs, loops, and mature miRNA regions of the groups A and B. The error bars were drawn with 95% confidence intervals. **f** The sequence and structure entropies of the ILs of groups, A and B. The pri-miRNAs were classified into 4 groups, as shown in the figure. **g** The sequence and structure entropy profiles of hsa-pri-mir-92a-1 orthologs containing IL3/6 (the left panel) and hsa-pri-mir-92a-1 orthologs containing IL3/3 (the right panel). The entropy value for each position was colored according to the color bar (right). The green arrowheads represent the cleavage sites of DROSHA.

In different animal species, DROSHA proteins share a similar domain arrangement. They all possess N-terminal proline-rich (P-rich), and arginine/serine-rich (R/S-rich) domains, a central domain (CED), and two tandem RNase III domains (RIIIDs; RIIIDa and RIIIDb), which are connected to a C-terminal dsRNA-binding domain (dsRBD) (Fig. 1a). The P-rich and R/S-rich domains of DROSHA vary in length and sequence among the different animal species. However, a study in humans showed that these domains are dispensable for the in vitro cleavage activity of DROSHA^8^. In contrast, the CEDs show conservation in their peptide sequences; for example, fly and worm CEDs share 47% and 28% identities, respectively, with the human homolog. Moreover, in humans, the CED is crucial for the cleavage function of DROSHA^9–12^. The RNase III domains (RIIIDs) are highly conserved in all DROSHA homologs, both regarding their sequences and catalytic mechanisms. For example, the amino acid sequences of the RIIIDs in human DROSHA are 65% and 45% identical to those in the fly and worm homologs, respectively. The two RIIIDs form an intramolecular heterodimer, creating a single cleavage center. Within the cleavage center, RIIIDa and RIIIDb cleave the 3p- and 5p-strands of pri-miRNAs, respectively^1,2,9–12^. However, it is still not known whether these two RIIIDs affect the cleavage activity of each other. The dsRBDs of animal DROSHAs show a high homology in their sequences. For example, the dsRBD polypeptide sequence of human DROSHA shares 61% and 41% amino acid sequence identity with the fly and worm DROSHAs, respectively. The dsRBD is essential for human DROSHA cleavage activity^9^ and interacts with mismatched GHG motifs (H being any nucleotide except G) in the lower stem of pri-miRNAs, thereby ensuring the accuracy and efficiency of DROSHA cleavage^13,14^. Even though dsRBD is known to be present in all the DROSHA homologs, whether it has a universal role in aiding the function of DROSHA in different animal species is still unknown.

Human DROSHA exhibits the 5p-strand single cleavage of a subset of pri-miRNAs that contain internal loops (ILs) in the lower stem^15^. The ILs have an inhibitory effect on the 3p-strand cleavage activity of DROSHA. This uncouples the double cleavages and helps DROSHA release the 5p-strand single cleavage products (Supplementary Fig. 1a). Changes in the size of the ILs of pri-miRNAs alter the 5p-strand single cleavage level, thereby controlling the efficiency of human DROSHA to produce pre-miRNAs^15^. However, it is not known how the ILs or other unknown RNA elements inhibit the 3p-cleavage ability of DROSHA and if DROSHAs of other animals also exhibit such single cleavage activity. In this study, we purified DROSHAs from flies and worms and demonstrated that the single cleavage is a common feature of all the tested DROSHAs. We also elucidated the molecular mechanism of the single cleavage by a series of mutagenesis experiments and pri-miRNA cleavage assays and showed that it involves two significant interactions between DROSHA and pri-miRNAs.

## Results

### The lower stem IL is a common feature of many pri-miRNAs in animals

We collected 10,895 pri-miRNA sequences from all 45 species deposited in MirGeneDB 2.0^16^ and folded them using the RNAfold^17^. An ILx/y (x + y > 2) is a single-stranded RNA (ssRNA) region in the lower stem of pri-miRNAs that contains x and y nucleotides (nts) on the 5p-strand and 3p-strand, respectively (Fig. 1b). Interestingly, we found that like human pri-miRNAs, numerous pri-miRNAs across all the animal species tested contain ILs in their lower stems. The frequencies of the IL-pri-miRNAs range between 23.75–70.99% in 45 organisms. For example, in *C*. *elegans* and *D*. *melanogaster*, the IL-pri-miRNAs are 70.99% and 42.22% of their pri-miRNAs, respectively (Fig. 1c, panel I). In addition, most ILs are located at 6–8 nt from the cleavage sites of DROSHA and have a size of 2/2 (Fig. 1c, panel II and III).

The ILs in the orthologous pri-miRNAs of a human pri-miRNA that share a similar size (i.e., number of nts) and the position relative to the cleavage sites of DROSHA with the IL of the human pri-miRNA were classified as being “similar.” We then calculated the percentage of orthologous pri-miRNAs containing a similar IL among the total orthologous pri-miRNAs of each human pri-miRNA (Fig. 1d). We found that 94 human IL-pri-miRNAs shared the similar IL with at least 2 other orthologous pri-miRNAs. We estimated the sequence and structure entropies (which indicate their variability) for different nucleotides in the similar-IL-pri-miRNAs and non-similar IL-pri-miRNAs. We found that the upper stem region of pri-miRNAs (an RNA duplex containing highly conserved miRNA sequences) had lower entropies for both the sequences and structures (Fig. 1e). In contrast, the loops of the pri-miRNAs, which are ssRNA regions and do not contain miRNA sequences, possessed relatively high structure and sequence entropies. Interestingly, we found that the structure entropies of the similar ILs were relatively low and much smaller than those of the loops and the non-similar ILs. This suggests that those similar ILs might be evolutionarily conserved and serve essential and common functions.

We then classified the IL-pri-miRNAs into four groups based on the structure and sequence entropies of their ILs. (I) the IL structures and sequences were conserved; (II) the IL structures conserved but their sequences not conserved; (III) the IL structures and sequences were not conserved; (IV) the IL structures not conserved but their sequences conserved (Fig. 1f). The high sequence entropy-low structure entropy IL-pri-miRNAs (group II: for example, hsa-pri-mir-21, see the right panel of Fig. 1g and Supplementary Fig. 1b) suggest that the ILs might have evolved to have constant structures while allowing the nucleotide sequences to be changed. We then demonstrated that the IL3/3 in hsa-pri-mir-21 caused hMP to produce more the single cleavage/double cleavage (sc/dc), while the smaller IL1/1 stimulated less the sc/dc. In addition, similar levels of sc/dc were obtained from cleaving two hsa-pri-mir-21 variants containing similar-sized ILs with different sequences (Supplementary Fig. 1c–e).

### DROSHAs of other animals also exhibit the single cleavage on their IL-containing pri-miRNAs

**Fig. 2:**
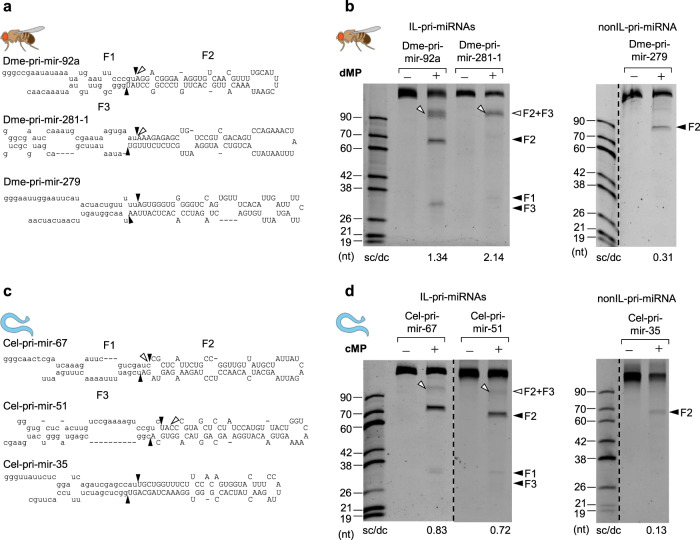
The single cleavage of the DROSHAs in animals. **a**, **c** Diagrams and sequences of pri-miRNAs from *D. melanogaster* and *C. elegans*. **b**, **d** Pri-miRNA cleavage assays. Three pmol of each pri-miRNA were incubated with 3 μL of fly or worm Microprocessor-bound IgG beads (50% slurry) for 2 h at 37 °C. dMP, *D. melanogaster* Microprocessor; cMP, *C. elegans* Microprocessor.

Since the human Microprocessor, hMP, shows the single cleavage activity on the IL-containing pri-miRNAs, we purified the Microprocessors of *D. melanogaster* (dMP) and *C. elegans (*cMP*)* (Supplementary Fig. 2a) and examined their single cleavage activity on IL-containing pri-miRNAs (Fig. 2a, c). As shown in Fig. 2b, d, both dMP and cMP produced longer-than-F2 products on the IL-containing substrates. These long products were the same size as the F2 + F3 fragment, which resulted from the cleavages of the 5pSC MP complexes that only retained the active site in their RIIIDb (Supplementary Fig. 2b). We then identified these long products by cloning and Sanger sequencing and confirmed they were the 5p-strand single cleavage products (Supplementary Fig. 2c). Consistent with hMP, dMP and cMP produced undetectable single cleavage products from the two tested non-IL pri-miRNAs (Fig. 2b, d). Therefore, the single cleavage activity appears to be a common feature of animal Microprocessors.

### The size of the ILs determines the level of single cleavage of animal DROSHAs

**Fig. 3:**
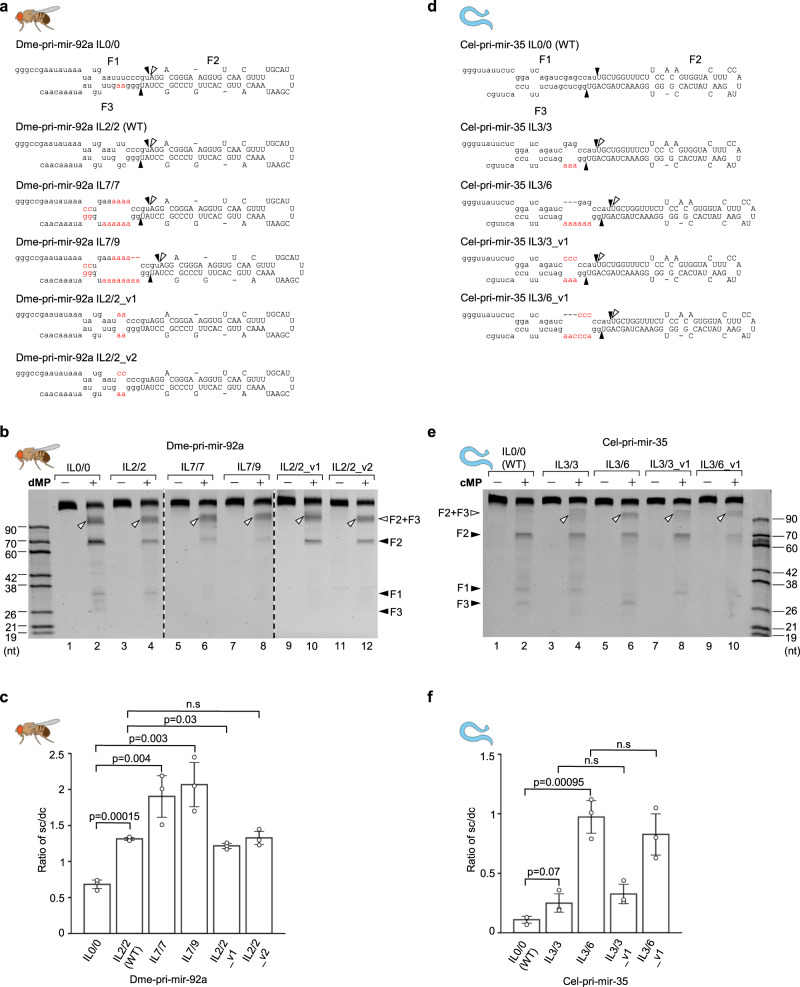
The ILs stimulate single cleavage of DROSHAs in animals. **a**, **d** Diagrams and sequences of pri-miRNAs and their variants. The mutated nt are in red. **b**, **e** Pri-miRNA cleavage assays. Three pmol of each pri-miRNA were incubated with 3 μL of Microprocessor-bound IgG beads for 2 h at 37°C. **c**, **f** The sc/dc ratio was calculated as the ratio of the single-cut (F2 + F3) to double-cut product (F2) for three repeated pri-miRNA cleavage results as shown in (**b**) and (**e**). The *p*-values of the two-tailed *t*-test for the sc/dc ratios estimated from three replicates were shown. The error bars represent SEM. dMP, *D. melanogaster* Microprocessor; cMP, *C. elegans* Microprocessor.

We next examined the effect of IL sizes in controlling the single cleavage of *D. melanogaster* and *C. elegans* DROSHAs. We generated four dme-pri-mir-92a variants and three cel-pri-mir-35 variants that contained different-sized ILs (Fig. 3a, d) and estimated the sc/dc of the MPs for each variant. We found a positive correlation of the sc/dc ratios and the increased sizes of ILs for both pri-miRNAs (Fig. 3b, lanes 2, 4, 6, 8; Fig. 3c; Fig. 3e, lanes 2, 4, 6; and Fig. 3f). These results were consistent with what had been previously demonstrated for several human pri-miRNAs, including hsa-pri-mir-92a-1, hsa-pri-mir-216a, hsa-pri-mir-204, and hsa-pri-mir-181a-1^15^. In addition, reduction in the size of ILs in other pri-miRNAs, cel-pri-mir-67, and cel-pri-mir-51, also decreased the sc/dc ratios of the cMP (Supplementary Fig. 3a–f). Besides, we also demonstrated that the sizes of ILs, but not their sequences, functioned in enhancing the sc/dc of DROSHAs (Fig. 3b, compare lanes 4 with 10, 12; Fig. 3e, compare lanes 4 with 8, and 6 with 10; and Fig. 3c, f). Our collective results indicate that the ILs in the lower stem (common in many animal species) stimulate the sc/dc of DROSHAs, and the size of the ILs determines the levels of sc/dc.

### The ILs cause the single cleavage of animal DROSHAs by disrupting the dsRBD and lower stem interaction

**Fig. 4:**
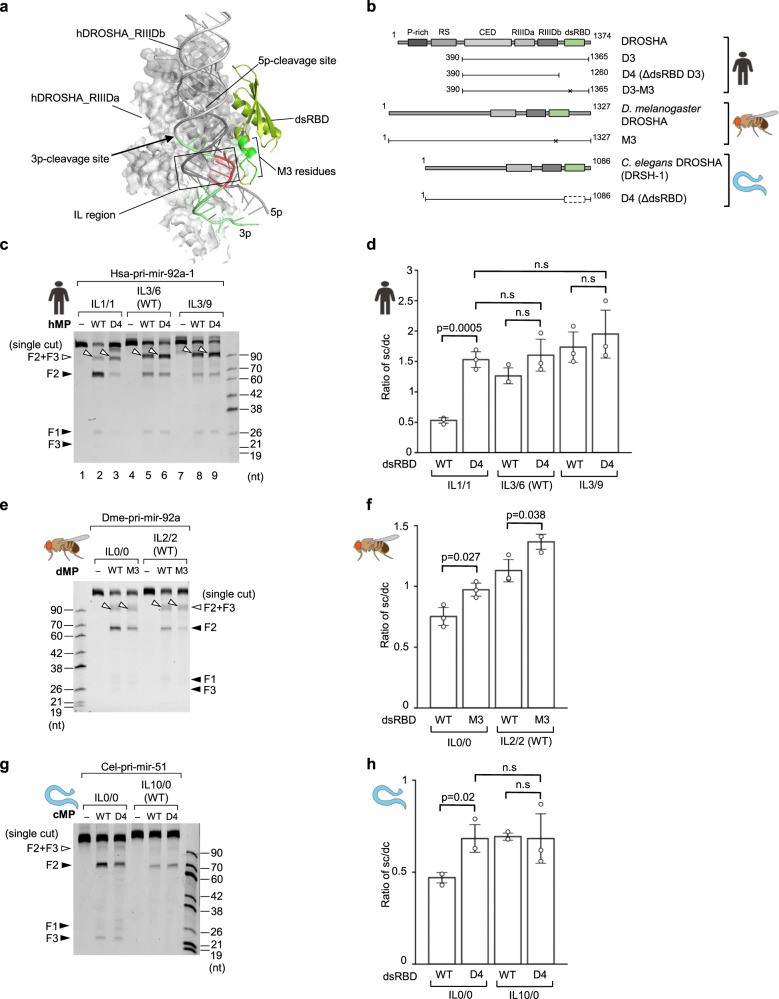
The ILs disrupt the interaction of dsRBD and lower stem of pri-miRNAs. **a** Hypothetical model to show the interruption of the dsRBD-lower stem interaction by the IL. The model was animated from the DROSHA-RNA structure (PDB: 6V5B). A similar result was also obtained from PDB: 6LXD. The dsRBD was shown in limon. The M3 residues and the IL regions were colored in green and red, respectively. The 3p-strand of the lower stem was shown in lime. **b** The DROSHA fragments were purified and used in this study. The numbers indicate the positions of amino acids at the N- and C-terminus of each protein fragment. The asterisks indicate the mutation sites. **c**, **e**, **g** Pri-miRNA cleavage assays. Five pmol of pri-miRNAs were incubated with 5 pmol of human WT or mutant Microprocessor (in **c**); 3 pmol of pri-miRNAs were incubated with 3 μL of fly or worm WT and mutant Microprocessor-bound IgG beads (in **e**, **g**) for 2 h at 37 °C. **d**, **f**, **h** The sc/dc ratio was calculated as the ratio of the single-cut (F2 + F3) to double-cut product (F2) band density for three repeated pri-miRNA cleavage results as shown in (**c,** **e**, **g**). The *p*-values of the two-tailed *t*-test for the sc/dc ratios estimated from three replicates were shown. The error bars represent SEM. hMP, human Microprocessor; dMP, *D. melanogaster* Microprocessor; cMP, *C. elegans* Microprocessor.

DROSHA uses the dsRBD to recognize and bind to the lower stem of pri-miRNAs^11,12,14^ (Fig. 4a). In addition, the ILs located in the lower stem diminish the 3p-strand cleavage ability of DROSHA. Therefore, any distortion in the lower stem (due to the ILs) might abolish its interaction with the dsRBD, and thus block the 3p-strand cleavage.

We compared the 5p-strand single cleavage levels of wild-type (WT) and dsRBD-deleted (D4) MP complexes (Fig. 4b and Supplementary Fig. 4a) and found that the latter significantly increased the ratio of 5p-strand single-to-double cleavages in hsa-pri-mir-92a-1 IL1/1 (Fig. 4c, lanes 2 and 3; and Fig. 4d). As hsa-pri-mir-92a-1 IL1/1 contained a small IL, the interaction between its lower stem and dsRBD was expected to be retained. These results indicate that a loss of the dsRBD causes the 5p-strand single cleavage. In agreement with previous observations, the large ILs were shown to highly enhance the level of 5p-strand single cleavage of the WT hMP (Fig. 4c, compare lanes 2 with 5 and 8; and Fig. 4d). In addition, the deletion of dsRBD did not extend the 5p-strand single cleavage further in the large IL-containing pri-miRNAs (i.e., hsa-pri-mir-92a-1 IL3/6 (WT) and IL3/9; Fig. 4c, compare lanes 5 with 6, and lanes 8 with 9; and Fig. 4d), suggesting that IL3/6 and IL3/9 were bulky enough to collapse the interaction between the IL and dsRBD. We also generated an M3 mutant of DROSHA (Fig. 4b and Supplementary Fig. 4a), which contained mutations in several dsRBD residues, critical for interacting with the mGHG motif in the lower stem of pri-miRNAs^14^. We found that this M3 mutant showed similar cleavage patterns for hsa-pri-mir-92a-1 IL1/1, IL3/6 (WT), and IL3/9 as the dsRBD-deleted MP (Supplementary Fig. 4b, c). We also obtained a similar result for hsa-pri-mir-181a-1 in Supplementary Fig. 4d, e. These results support that a disruption of the dsRBD-lower stem interaction is a cause of the 5p-strand single cleavage.

We then examined the effect of dsRBD on the single cleavage activity of dMP and cMP by performing similar mutagenesis and cleavage assays as described for hMP. Similar to hMP, we found that the dsRBD-mutant dMP (dMP-M3) and dsRBD-mutant cMP (cMP-D4) also increased the sc/dc ratios on non or small IL-containing dme-pri-mir-92a and cel-pri-mir-51 variants (Fig. 4b, e–h).

### dsRBD supports the 3p-strand cleavage of animal DROSHAs

**Fig. 5:**
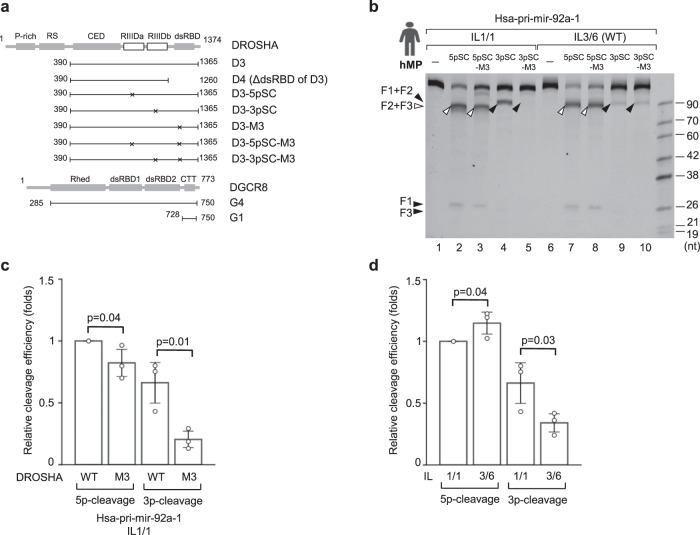
dsRBD supports the 3p-strand cleavage of animal DROSHAs. **a** The DROSHA and DGCR8 fragments were purified and used in this figure. The numbers indicate the positions of amino acids at the N- and C-terminus of each protein fragment. The asterisks indicate the mutation sites. Rhed, RNA heme-binding domain; CTT, C-terminal tail. Pri-miRNA cleavage assays. **b** Five pmol of pri-miRNAs were incubated with 5 pmol of human WT or mutant Microprocessor for 2 h at 37 °C. **c**, **d** The cleavage efficiency of the WT and mutant Microprocessor in (**b**). The cleavage efficiency was estimated as the ratio of the F2 + F3 or F1 + F2 to the original substrates. The *p*-values of the two-tailed *t*-test for the relative cleavage efficiency estimated from three replicates were shown. The error bars represent SEM. hMP, human Microprocessor.

We next dissected the functions of dsRBD in supporting the 5p- and 3p-cleavage of DROSHA individually. First, we generated D3-5pSC and D3-3pSC proteins, which contained mutations in the catalytic centers of RIIIDa (D3-5pSC, glutamic acid in the 1,045^th^ position in RIIIDa was mutated into glutamine) or RIIIDb (D3-3pSC, glutamic acid in the 1,222^nd^ position in RIIIDb was mutated into lysine). As a result, D3-5pSC and D3-3pSC cleaved pri-miRNAs only on their 5p-strand and 3p-strand, respectively. We then introduced M3 mutations in both proteins to generate D3-M3-5pSC and D3-M3-3pSC (Fig. 5a and Supplementary Fig. 5a). Interestingly, we showed that the M3 mutations substantially impaired the 3p-cleavage of DROSHA in hsa-pri-mir-92a-1 IL1/1 and hsa-pri-mir-181a-1 IL2/2, but it only minimally compromised the 5p-cleavage of DROSHA in the same substrate (Fig. 5b, compare lanes 4 with 5, and lanes 2 with 3; Fig. 5c; and Supplementary Fig. 5b–f). The large IL-containing hsa-pri-mir-92a-1 IL3/6 and hsa-pri-mir-181a-1 IL2/5 were more invulnerable to the M3 mutations than the small IL-containing pri-miRNAs (Fig. 5b, compare lanes 7 with 8, and lanes 9 with 10; and Supplementary Fig. 5b–f). These data indicate that the dsRBD is more crucial for the 3p-cleavage of DROSHA than it is for the 5p-cleavage. In addition, and consistent with previous observations^15^, the large IL significantly impaired the 3p-cleavage (Fig. 5d). Therefore, the ILs likely induce the 5p-strand single cleavage of DROSHA by preventing dsRBD from supporting the 3p-cleavage.

### The conserved functions of a 3p-strand cleavage-supporting helix (3pCSH) in ensuring the double cleavage of animal DROSHAs

**Fig. 6:**
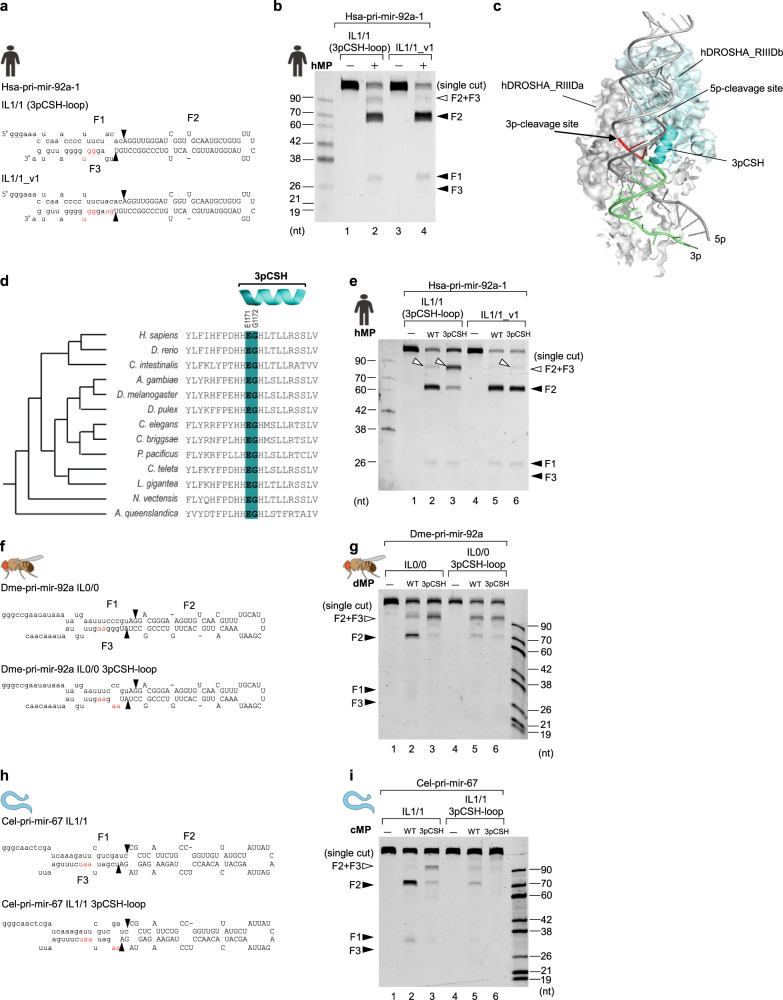
The conserved functions of 3p-strand cleavage-supporting helix (3pCSH) in ensuring the double cleavage of animal DROSHAs. **a**, **f**, **h** Diagrams and sequences of pri-miRNAs and their variants. The mutated nt are in red. **b**, **e**, **g**, **i** Pri-miRNA cleavage assays. Five pmol of each pri-miRNA were incubated with 5 pmol of human WT or mutant Microprocessor (in **b**, **e**), or 3 pmol of each pri-miRNAs were incubated with 3 μL of fly or worm WT and mutant Microprocessor-bound IgG beads (in **g**, **i**) for 2 h at 37°C. **c** The DROSHA-RNA interaction model was animated from PDB: 6V5B (a similar model was also obtained from PDB: 6LXD). The 3p-CSH was colored in cyan. The amino acids of the 3p-CSH and the RNA elements, which might make a potential interaction with each other, were shown in deep-teal and red, respectively. The 3p-strand of the lower stem was shown in lime. **d** The conservation of the 3p-CSH in animals. The polypeptides of the 3pCSH region of DROSHA from 13 organisms were aligned using Clustal Omega^31^.

We noticed that the hMP produced some single cleavage products from hsa-pri-mir-92a-1 IL1/1, which contained a small IL. We, therefore, hypothesized that RNA elements in hsa-pri-mir-92a-1 other than ILs might also affect the 3p-cleavage. Consequently, we investigated two mismatches (in the −3 to −4 positions) in the lower stem of this pri-miRNA. We found that the hMP no longer generated the single cleavage from hsa-pri-mir-92a-1 IL1/1_v1 that contained base pairs in the −3–4 region (Fig. 6a, b). This suggests that these mismatches might interrupt another interaction between DROSHA and RNA, critical for the 3p-strand cleavage.

We analyzed the structures of DROSHA-RNA^11,12^ and found that three residues (i.e., H1170, E1171, and, G1172), located at the N-terminus of the alpha-helix (amino acids 1170–1182; called a 3p-strand cleavage-supporting helix, 3pCSH), might make contact with the base-pairs in the −3 to −4 positions (or so-called 3pCSH region) (Fig. 6c). We aligned the polypeptides of DROSHA from 13 species and found that three amino acids at the N-termini of their 3pCSHs were conserved (Fig. 6d). We mutated two (E1171 and G1172) of these three amino acids into alanines and purified the mutant hMP (hMP-3pCSH; Supplementary Fig. 6a, b). As shown in Fig. 6e and Supplementary Fig. 6c–g, hMP-3pCSH generated several single cleavage products from hsa-pri-mir-92a-1 IL1/1_v1 and increased the level of single cleavage products from hsa-pri-mir-92a-1 IL1/1 3pCSH-loop and hsa-pri-mir-92a-1 IL1/1 3pCSH-loop 3MM. These results indicate that 3pCSH mutations in human DROSHA and mismatches in the 3pCSH region of pri-miRNAs partially disrupt the interaction between the 3pCSH and 3pCSH region, resulting in the 5p-strand single cleavage.

We next generated similar mutations in the 3pCSH of cMP and dMP (Supplementary Fig. 6a, b), or mismatches in the 3pCSH region of pri-miRNAs and examined the activity of the mutant enzymes. Again, we obtained similar results showing that the mismatches in the 3pCSH region of pri-miRNAs or mutations in the 3pCSH of enzymes caused more single cleavage of pri-miRNAs (Fig. 6f–i and Supplementary Fig. 7a–h).

## Discussion

Several critical features of pri-miRNAs influence the cleavage activity of DROSHA^8,13,14,18–23^. For example, the basal UG motif (at positions −14 and −13 on the 5p strand) enhances the cleavage efficiency of DROSHA^8,18^. The UGU and GUG motifs, located at the apical junction on the 5p-strand (termed the apical UGU motif), also enhance the cleavage activity of DROSHA by strengthening the interaction between DGCR8 and the apical loop^8,18,24,25^. The CNNC sequence (N is any nucleotide) in the flanking region of the 3p-strand functions with SRSF3 and thus helps recruit DROSHA to the basal junction, thereby stimulating the efficient cleavage of pri-miRNAs^18,19^. The mismatched GHG motif (mGHG motif; H being any nucleotide except G), located in the lower stem of pri-miRNAs, sharpens the pri-miRNA cleavage sites by DROSHA^13,14^. The midBMW_1012 located in positions 10–12 from DROSHA’s cleavage site in the upper stem enhances the cleavage of pri-miRNA by hMP^22,23^. Among these various previously identified pri-miRNA features, the mGHG motif and the midBMW_1012 seem to be conserved in bilaterian organisms (except the sea snail for mGHG)^13,14,18,22,23^. We previously revealed that the ILs located in the lower stem of pri-miRNAs might affect the cleavage efficiency of DROSHA^15^. This study found that ILs are widely present in the pri-miRNAs of bilaterian organisms (albeit with different frequencies). Interestingly, many identified ILs, which contain the different nt sequences, share the similar structures and positions in the pri-miRNA orthologs, suggesting that these ILs might be an evolutionarily conserved structural RNA element.

**Fig. 7:**
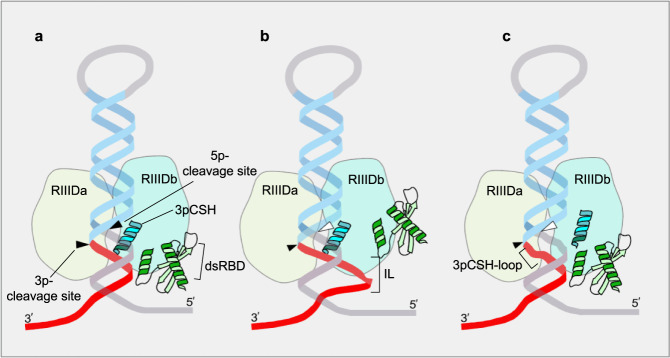
The conserved single cleavage mechanism of animal DROSHA enzymes. **a**–**c** The 3p-strand cleavage of DROSHA RIIIDa is strengthened by two interactions (**a**), RIIIDb_3pCSH-RNA and dsRBD-RNA. The IL (**b**) and 3pCSH-loop (**c**) interrupt the two interactions above, resulting in the single cleavage by DROSHA.

In the previous study^15^, we found that human DROSHA exhibits the single cleavage activity on the 5p-strand (and not the 3p-strand) of many of the pri-miRNAs tested. This suggests that the cleavage of the 5p-strand by DROSHA is more consistent than that of the 3p-strand. Perhaps, the 5p-strand cleavage, which the RIIIDb of DROSHA executes, is strengthened by its central domain (CED), essential for its enzyme activity, or by a robust intrinsic interaction between RIIIDb and the 5p-strand of pri-miRNAs. In contrast, cleavage of the 3p-strand, which RIIIDa of DROSHA conducts, seems more vulnerable. In this study, we discovered that the 3p-strand cleavage by RIIIDa is supported by two protein-RNA interactions, dsRBD-RNA and 3pCSH-RNA. Any distortion that occurs in these interactions, such as by the presence of IL or/and 3pCSH-loop, might cause the single cleavage by DROSHA (Fig. 7a–c). Controlling the single cleavage levels might be an important regulatory mechanism to control the cellular expression of miRNAs.

The findings in this study showed that the effect of the same-sized ILs is not the same for the different pri-miRNA backbones, suggesting that other RNA elements in pri-miRNAs might also contribute to the single cleavage; for example, the nucleotide sequences at the cleavage sites. In addition, the mutations in the 3pCSH of human DROSHA still caused an increase in the single cleavage of 3pCSH-loop-containing pri-miRNAs, whereas those mutations of the 3pCSH in DROSHA from flies and worms did not (Fig. 6e, g, i; Supplementary Fig. 6c–e and Supplementary Fig. 7). This suggests that the mutant 3pCSH in human DROSHA might still retain some RNA binding-affinity or human DROSHA might use its other domains to interact with the 3pCSH region. Furthermore, the DGCR8 dimer binds to pri-miRNA asymmetrically, which might contribute to determining the single cleavage. Many protein cofactors or RNA modifications might also control the single cleavage activity of DROSHA by influencing the structure of the IL and 3pCSH-loop or by affecting the interaction between DROSHA RIIIDa and RNA.

The uncoupling of double cleavages in RNase III was first found in *E. coli*^26–28^. Single and double cleavages in the same hairpin were the reason for the difference in protein expression observed in hairpin-containing mRNAs. Our findings suggest that DROSHAs in higher organisms might use single cleavage activity in different types of RNA, and identifying the single cleavage substrates of DROSHA in humans and other organisms will be necessary for future investigations.

## Methods

### Analysis of the sequence and structure conservation of ILs

We collected 10,895 pri-miRNA sequences (20-nt extending from both ends of pre-miRNA sequences) from 45 all species available in MirGeneDB 2.0^16^. We removed 975 duplicated pri-miRNAs that shared the identical IDs in miRBase.org^29^ and then predicted the secondary structures of the remaining pri-miRNAs using RNAfold^17^. 290 pri-miRNAs containing the multiple loops were also excluded. An ILx/y is an internal loop in the lower stem of pri-miRNA if it satisfies the following conditions. (1) It is an ssRNA region located in the lower stem of pri-miRNAs; (2) The first position of the IL is from −3 to −10 on the 5p-strand, or from −1 to −8 on the 3p-strand (Fig. 1b); (3) x + y > 2, in which x, y are the numbers of nt on the 5p- and 3p-strands of the IL, respectively. We then identified 148 IL-pri-miRNAs in humans and 3,159 IL-pri-miRNAs in 44 other species. The positions and types of ILs in each species were characterized (Supplementary Data 1).

We collected the pri-miRNA orthologs, described in the previous paper^30^ and deposited in MirGeneDB 2.0^16^, from 44 other species for each of 129 human IL-pri-miRNAs. The remaining 19 human IL-pri-miRNAs did not have orthologs in other species. We then identified ILs in those orthologous pri-miRNAs. If an ILx/y from an orthologous pri-miRNA of any species is located in a similar position (±1 nt difference allowed) and has a similar size (similar x/y) with that in a human pri-miRNA, it is considered as “the similar IL”. Then, the orthologous pri-miRNAs of each human pri-miRNA were classified into 2 groups, the “similar IL” orthologous pri-miRNAs (Group A) and “non-similar IL” orthologous pri-miRNAs (Group B) (Supplementary Data 2). Group A and B must have at least 1 orthologous pri-miRNA.

We converted the dot-blanket structures obtained from RNAfold to custom-designed symbols such that each nt was labeled with one of the five following letters: F (unpaired nt in the 5p-flanking fragment); T (unpaired nt in the 3p-flanking fragment); M (match); I (mismatch); and L (loop). Then, we performed multiple alignments for the sequences and structures of the human IL-pri-miRNAs and their orthologous pri-miRNAs in group A or group B using Clustal Omega^31^. Based on the scores resulting from the sequence and structure alignments, we estimated the Shannon entropy for the sequence and structure of each nt as follows. H = $-\mathop{\sum}\limits_{x}P(x){{\log }}_{2}(P(x))\,$ such that $x$: 1 in 4 nt (for sequence entropy) or 1 in 5 types of the positional structures (for structure entropy), and P($x$) is the fraction of $x$. The sequence or structure entropy of a specific position showed the variability of this position. For example, the lower sequence entropy indicated that the nt in this position was more highly conserved.

We also calculated the structure and sequence entropies for the ILs, loops, and mature miRNA regions for groups A and B of each human IL-pri-miRNA. The entropy for each area was calculated as the average of all nt in this region. We used 0.1 as the cutoff value for structure and sequence entropies and classified the human IL-pri-miRNAs into four groups: (I) The IL structures and sequences were conserved; (II) the IL structures were conserved, but their sequences were not conserved; (III) the IL structures and sequences were not conserved; IV) the IL structures were not conserved, but their sequences were conserved (Fig. 1f).

### Construction of protein expression plasmids

Total RNAs of *C. elegans* (whole-body) and *D. melanogaster* (ovary) were received from Dr. Ho Yi Mak and Dr. Yan Yan (Hong Kong University of Science and Technology). We synthesized cDNAs using these total RNAs and Oligo (dT) primer (IDT). Next, the coding sequences of DROSHA and DGCR8 in *C. elegans* (DROSHA and PASH-1) and *D. melanogaster* (DROSHA and PASHA) were PCR-amplified using specific forward and reverse primers for each gene as listed in Supplementary Table 1. The coding sequences of DROSHA orthologs were cloned into the pXab vector^8^, generating the pXab-dme-DROSHA and pXab-cel-DROSHA plasmids. As a result, the DROSHA protein was fused with the protein G at its C-terminus. The coding sequences of DGCR8 orthologs were inserted into the pXG vector^8^, generating the pXG-dme-PASHA and pXG-cel-PASH-1 plasmids. In the cloned plasmids, the DGCR8 protein was fused with GFP and 10x His-tag at its C-terminal. The primer sequences for amplifying the vector for each gene were provided in Supplementary Table 1. The mutant protein-expressing plasmids were constructed based on the WT protein-expressing plasmids. The information of the cloning primers were shown in Supplementary Table 2.

### Purification of recombinant proteins

The human Microprocessor complex (hMP) was reconstituted by mixing the purified D3-G1 complex with G4 dimer. To purify the *D. melanogaster* Microprocessor complex (dMP), the pXab-dme-DROSHA and the pXG-dme-PASHA plasmids were transfected together into 40 dishes (100 mm in diameter) of HEK293E cells. The transfected cells were collected after 3 days. Next, the cell pellets were dissolved in 40 ml T500 lysis buffer (20 mM Tris-HCl (pH 7.5), 500 mM NaCl, and 4 mM β-mercaptoethanol), supplemented with 2 μg/ml RNase A and protease inhibitor cocktail. The dissolved cells were then sonicated and centrifuged, after which the clear lysate was obtained and mixed with 5 ml of pre-equilibrated Ni-NTA resin (Thermo Fisher). The mixture was rotated for 30 min at 4 °C to allow the complex to bind to the resin. The protein-bound resin was washed with 90 mL of T0 and T2000 containing 20 mM Tris-HCl (pH 7.5), 4 mM β-mercaptoethanol plus 0 and 2000 mM NaCl, respectively, and finally with 90 mL of T500 supplemented with 50 mM imidazole. The beads were eluted with T500 containing 300 mM imidazole. The eluates were pooled and added with 4 mM DTT before being bound to the pre-equilibrated IgG sepharose at 4 °C for 4 h. The protein-bound IgG beads were washed with T500 supplemented with 4 mM DTT three times and resuspended in T500, 4 mM DTT, and 20% glycerol for −80°C storage. To purify the *C. elegans* Microprocessor (cMP), the pXab-cel-DROSHA and the pXG-cel-PASH-1 plasmids were co-transfected into 40 dishes HEK293E cells. The purification of cMP was performed similarly as described for dMP.

### The preparation of pri-miRNA substrates

The DNA fragments encoding pri-miRNAs were PCR-amplified from genomic DNA using a pair of T7 promoter-containing forward and reverse primers. The worm and fly genomic DNA were received from Dr. Ho Yi Mak and Dr. Yan Yan (the Hong Kong University of Science and Technology, Hong Kong, China). For the difficult-to-amplify fragment, the DNAs were synthesized from the extension of two long primers that contained the T7 promoter and pri-miRNA sequences. The primers and amplification methods used for each pri-miRNA were detailed in Supplementary Table 3. Two hundred ng of amplified T7-containing DNAs were used in a 20 μL reaction mixture of the in vitro transcription (IVT) (the MEGAscript T7 Kit, (Invitrogen, AMB13345). The synthesized RNAs were gel-eluted and precipitated using isopropanol (IPA). The precipitated RNAs were then dissolved in distilled water (DW) and stored at −80 °C for later use.

### In vitro pri-miRNA cleavage assays

Five pmol of each human pri-miRNA and 3 pmol of each fly or worm pri-miRNA were added in the pri-miRNA cleavage assays. Five pmol of hMP or 3 μL of dMP (or cMP)-bound IgG beads (50% slurry) were then added in the 10 μL of the reaction mixture containing 50 mM Tris-HCl (pH 7.5), 150 mM NaCl, 2 mM MgCl_2_, 10% glycerol, 0.2 μg/μL BSA, 1 mM DTT and 2 U SUPERase•In™ RNase Inhibitor (Thermo Fisher). The 0.3 μM of RNAs and 0.5 μM of the enzymes were used in the reaction mixture. In the titration cleavage assays, the amounts of enzymes were indicated in the figure legends. The reaction was carried out at 37 °C for 2 h. After that, 10 μL of 2× TBE-urea sample buffer were added to stop the reaction. The stopped reaction mixture was immediately chilled on ice and supplemented with 20 μg proteinase K (Thermo Fisher). The proteinase K-supplemented mixture was incubated at 37 °C for 15 min, and then at 50 °C for 15 min, and finally at 95 °C for 10 min. Then the processed RNAs were loaded onto a pre-run 12% urea-PAGE in a 1× TBE buffer. The gel was run at 300 V for 40 min and was later stained with SYBR™ Green II RNA Gel Stain (Invitrogen, S7564) for 10 min. The stained gels were imaged using the Bio-Rad Gel Doc XR+ system. The intensities of the RNA bands detected in the gels were measured using Image Lab 3.0.

### Sequencing of single cleavage products

The long cleavage products generated from the pri-miRNA cleavage assays were extracted and purified by IPA. The purified RNAs were ligated with a 5′-adapter (RA5-4N, GUU CAG AGU UCU ACA GUC CGA CGA UCN NNN) using T4 RNA ligase I (NEB, M0204L). The ligated RNAs were reverse transcribed using Superscript IV reverse transcriptase (Invitrogen, 18090050) and the RT primer specific to the 3′-segment of each pri-miRNA. The resulting cDNAs were PCR-amplified using a pair of sequencing primers, RP1 (AAT GAT ACG GCG ACC ACC GAG ATC TAC ACG TTC AGA GTT CTA CAG TCC GA), and each of the specific primers, RPIx. The DNAs were Sanger-sequenced using F-RP1 primer (AAT GAT ACG GCG ACC ACC GAG ATC TA) and specific reverse primer.

### Statistics and reproducibility

The two-tailed *t*-test was used to calculate the *p*-values of the sc/dc ratios estimated from three biological replicates.

## Supplementary information

Supplementary Information

Description of Additional Supplementary Files

Supplementary Data 1

Supplementary Data 2

Supplementary Data 3

## Data Availability

Pri-miRNA sequences were obtained from MirGeneDB 2.0. PDB: 6V5B and 6LXD were used for structural modeling. The Sanger sequences of the single cleavage product have been deposited at the Mendeley Data Repository (doi: 10.17632/czzbnwpzxz.1). Plasmids generated were deposited to Addgene (pXab-cel-DROSHA (178539), pXG-cel-PASH-1 (178540), pXab-dme-DROSHA (178541), and pXG-dme-PASHA (178542)). Source data for the graphs and charts in Fig. 1d–e, 3c, f, 4d, f, h, 5c‒d; and Supplementary Figs. 1e, 3c, f, 5e‒f,6c, g, 7a, b, 7e, h are presented as Supplementary Data 3. Uncropped gel images are in Supplementary Figs. 8–13. All other data are available from the corresponding author upon reasonable request.
