# Supplementary material for: The conserved single-cleavage mechanism of animal DROSHA enzymes
Source: Commun Biol. 2021 Nov 25;4:1332. doi: 10.1038/s42003-021-02860-1 (PMC8616936; doi:10.1038/s42003-021-02860-1)
Supplement: Supplementary file 1 — Supplementary Information [file 42003_2021_2860_MOESM1_ESM.pdf]

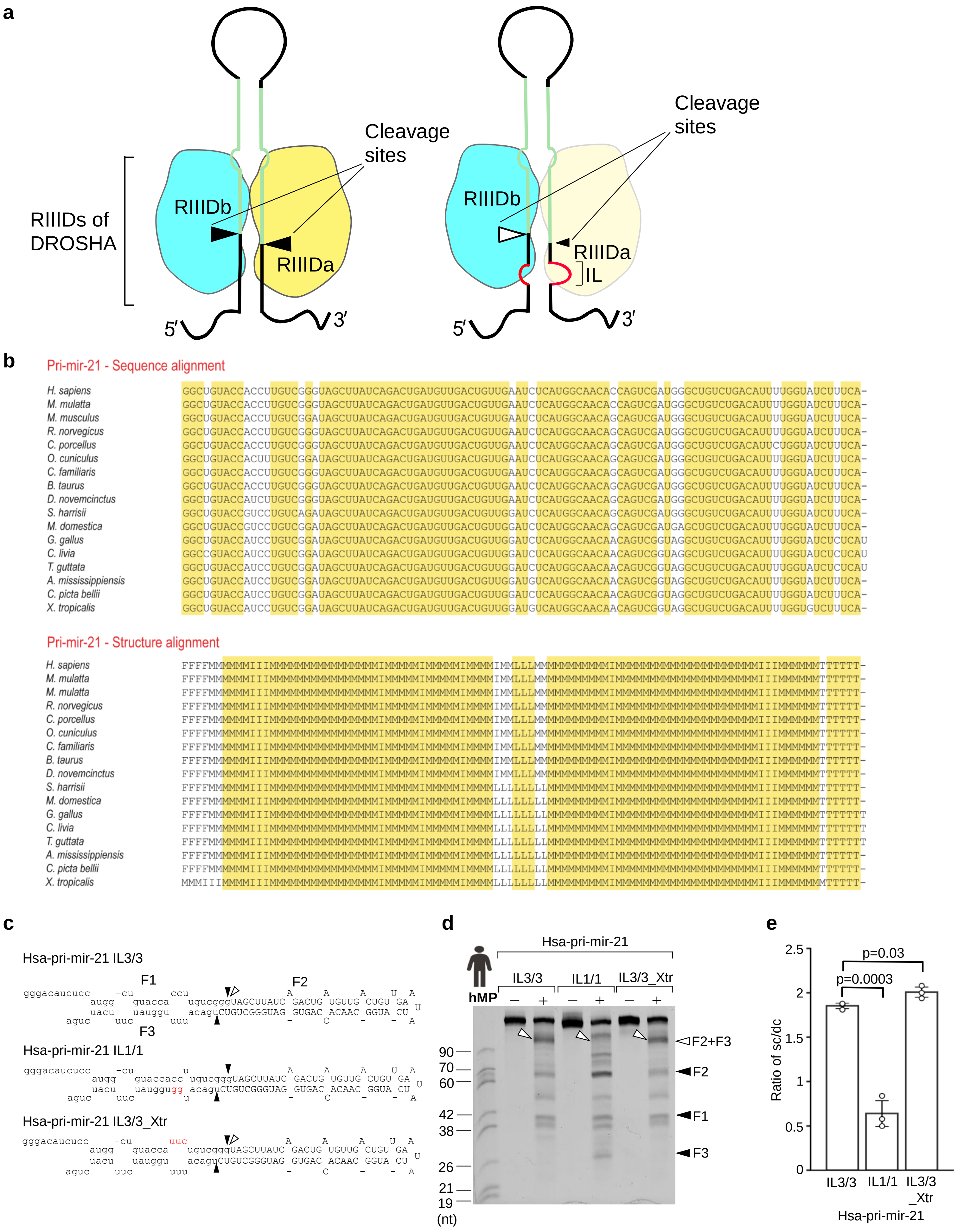

Supplementary Fig. 1 The internal loop (IL) is a common feature of animal pri-miRNAs. a) The internal loop (IL) induces the single cleavage activity of human DROSHA. The RIIIDa and RIIIDb of DROSHA are in yellow and cyan, respectively. The miRNA region is in light green. The black arrowheads indicate the DROSHA's cleavage sites. The IL is in red. **b**) The representative alignments of hsa-pri-mir-21 orthologs' sequences (the upper panel) and structures (the lower panel). The positions conserved in all orthologs were highlighted in yellow. **c**) Diagrams and sequences of hsa-pri-mir-21 and its variants. The mutated nt are in red. **d**) Pri-miRNA cleavage assays. Five pmol of pri-miRNAs were incubated with 5 pmol of human Microprocessor (hMP) for 2 h at 37ºC. **e**) The sc/dc ratio was calculated as the ratio of the single-cut (F2+F3) to double-cut product (F2) band density for three repeated pri-miRNA cleavage results as shown in (d). The p-values of the two-tailed t-test for the sc/dc ratios estimated from three replicates were shown. The error bars represent SEM.

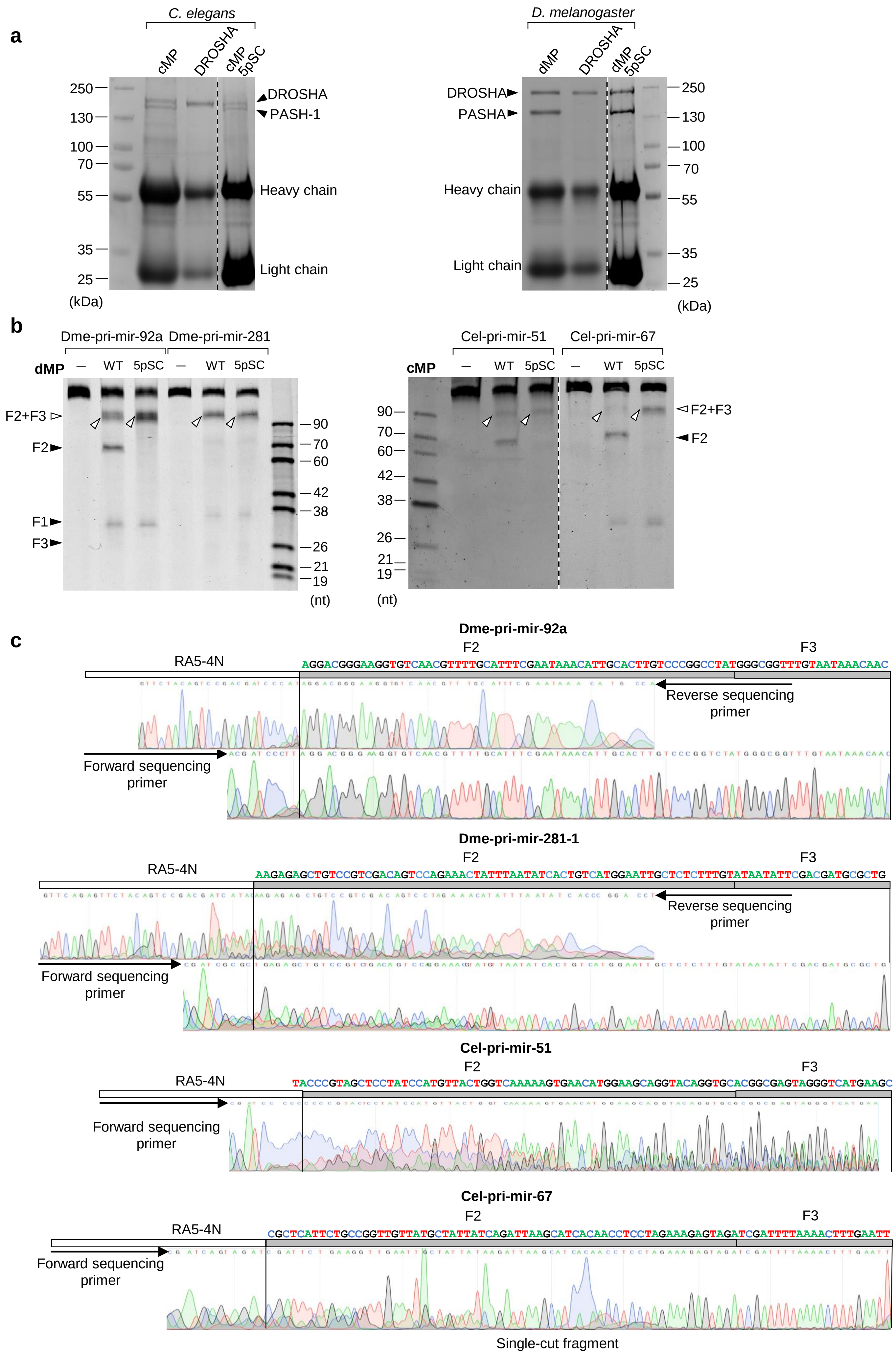

Supplementary Fig. 2 Single cleavage of DROSHAs in animals. a) The quality of the purified Microprocessor and DROSHA proteins was assessed in SDS-PAGE. The heavy and light chains were the components of IgG-sepharose used in protein purification. **b**) Pri-miRNA cleavage assays. Three pmol of pri-miRNAs were incubated with 3 µL of WT or mutant dMP (or cMP)-bound IgG beads for 2 h at 37ºC. **c**) The Sanger sequencing results of single cleavage products (F2+F3) resulted from the pri-miRNA cleavage assays by the Microprocessor shown in Fig. 2b and 2d. RA5-4N was the adapter sequence used in RNA cloning. The F2+F3 sequences were sequenced by either reverse or forward primers shown in this figure. dMP, *D. melanogaster* Microprocessor; cMP, *C. elegans* Microprocessor.

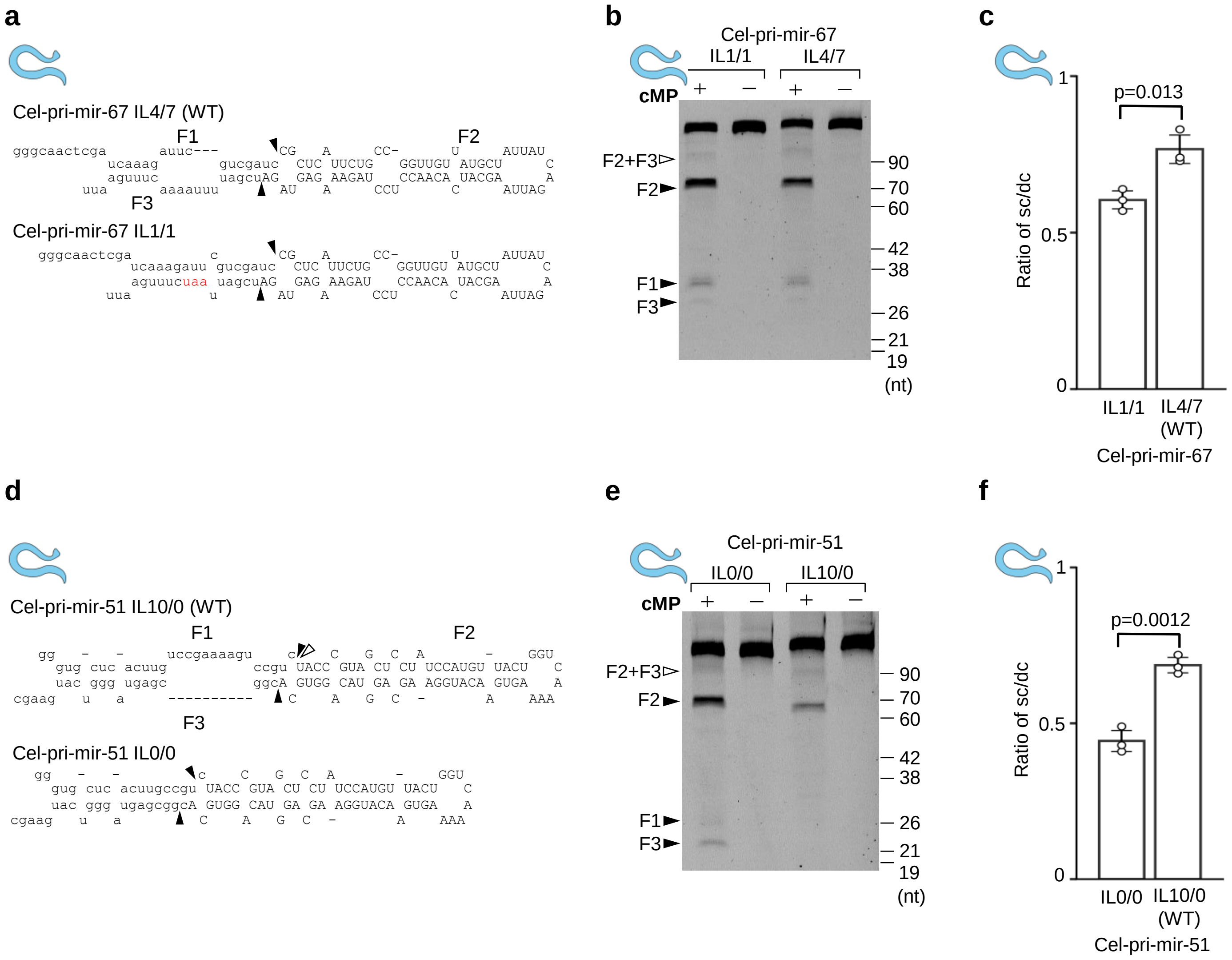

Supplementary Fig. 3 The ILs stimulate single cleavage of DROSHAs in animals. a, **d**) Diagrams and sequences of cel-pri-mir-67, cel-pri-mir-51, and their variants. The mutated nt are in red. **b**, **e**) Pri-miRNA cleavage assays. Three pmol of each pri-miRNA were incubated with 3 µL of Microprocessor-bound IgG beads for 2 h at 37ºC. **c, f**) The sc/dc ratio was calculated as the ratio of the single-cut (F2+F3) to double-cut product (F2) for three repeated pri-miRNA cleavage results as shown in (b). The p-values of the two-tailed t-test for the sc/dc ratios estimated from three replicates were shown. The error bars represent SEM. cMP, *C. elegans* Microprocessor.

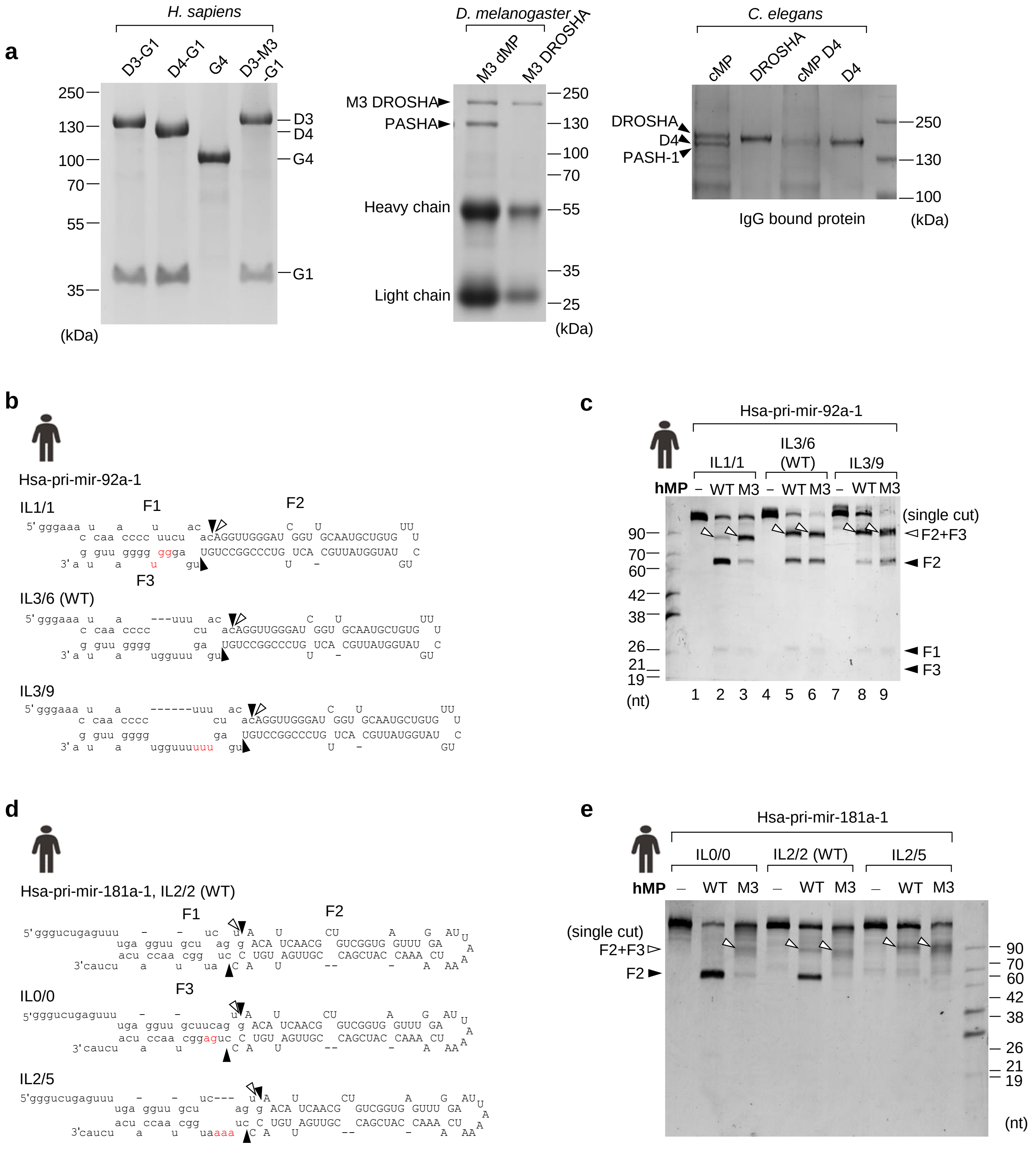

Supplementary Fig. 4 The ILs disrupt the interaction of dsRBD and the lower stem of pri-miRNAs. a) The quality of the purified Microprocessor and DROSHA proteins was assessed in SDS-PAGE. The heavy and light chains were the components of IgG-sepharose used in protein purification. **b**) Diagrams and sequences of human hsa-pri-mir-92a-1 and its variants. The mutated nt are shown in red. **c)** Pri-miRNA cleavage assays. Five pmol of pri-miRNAs were incubated with 5 pmol of Microprocessor for 2 h at 37ºC. **d**) Diagrams and sequences of human hsa-pri-mir-181a-1 and its variants. The mutated nt are shown in red. **e**) Pri-miRNA cleavage assays. Five pmol of pri-miRNAs were incubated with 5 pmol of Microprocessor for 2 h at 37ºC. hMP, human Microprocessor.

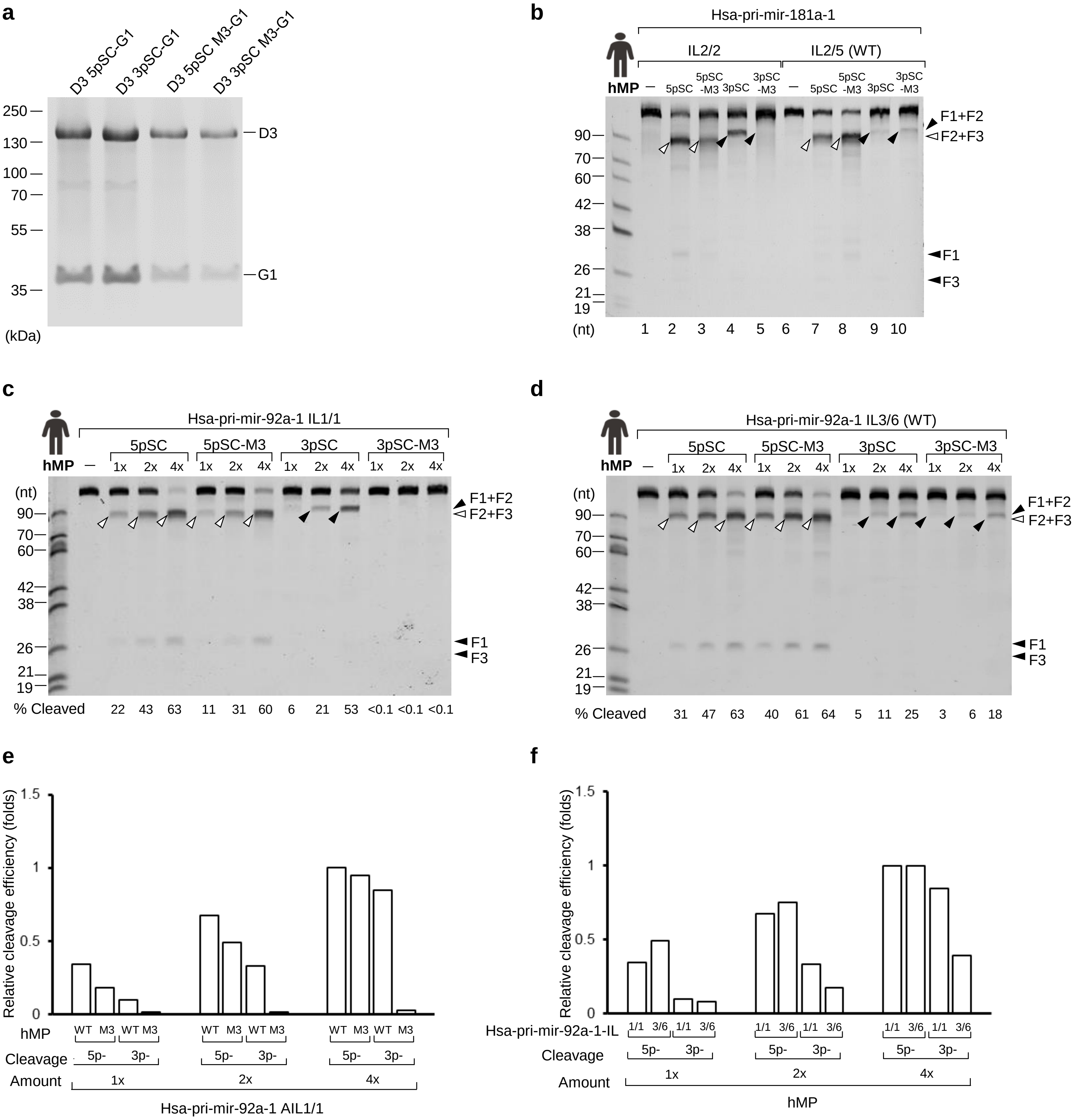

Supplementary Fig. 5 dsRBD supports the 3p-strand cleavage of human DROSHAs. a) The quality of the purified Microprocessor proteins was assessed in SDS-PAGE. **b**) Pri-miRNA cleavage assays. Five pmol of pri-miRNAs were incubated with 5 pmol of Microprocessor for 2 h at 37ºC. **c, d**) Pri-miRNA titration cleavage assays of hMP. The three different amounts of hMP (1.25, 2.5, and 5 pmol) were incubated with 5 pmol pri-miRNAs (hsa-pri-mir-92a-1 IL1/1 in (c) and IL3/6 in (d)). **e, f**) The sc/dc ratio was calculated as the ratio of the single-cut (F2+F3) to double-cut product (F2) for the pri-miRNA cleavage results as shown in (c) and (d). hMP, human Microprocessor.

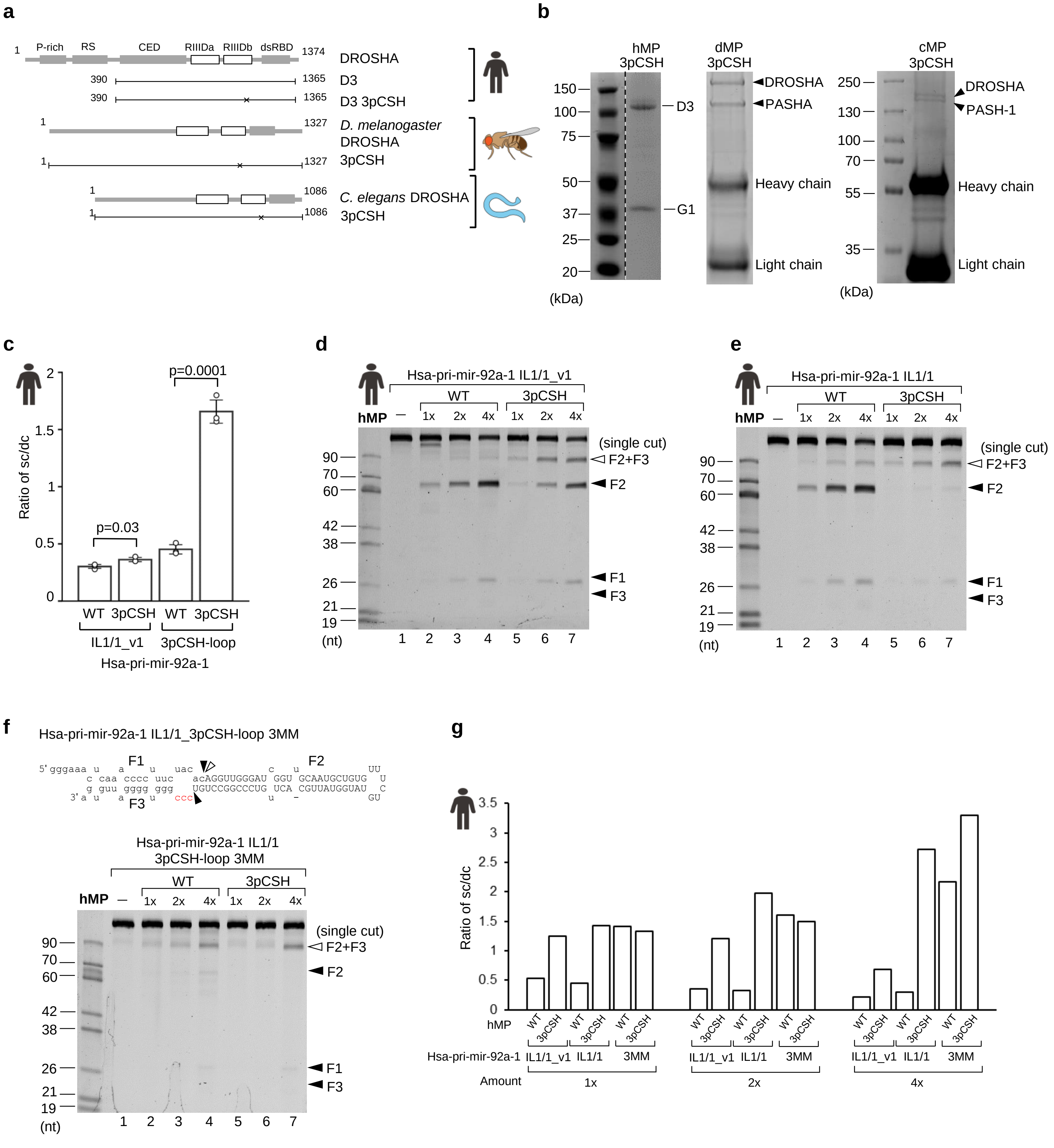

Supplementary Fig. 6 3pCSH is essential for the 3p-cleavage of animal DROSHAs. a) Diagram of protein constructs used in Fig. 6. **b**) The quality of purified 3pCSH-mutated Microprocessors in humans (hMP-3pCSH), flies (dMP-3pCSH), and worms (cMP-3pCSH) was assessed in SDS-PAGE. **c**) The sc/dc ratio was calculated as the ratio of the single-cut (F2+F3) to double-cut product (F2) band density for three repeated pri-miRNA cleavage results as shown in Fig. 6e. The p-values of the two-tailed t-test for the sc/dc ratios estimated from three replicates were shown. The error bars represent SEM. **d**, **e, f**) Pri-miRNA titration cleavage assays of hMP. The three different amounts of hMP (WT and 3pCSH) (0.625, 1.25, and 2.5 pmol) were incubated with 5 pmol of pri-miRNAs. **g**) The sc/dc ratio was calculated as the ratio of the single-cut (F2+F3) to double-cut product (F2) for the pri-miRNA cleavage results as shown in (d–f).

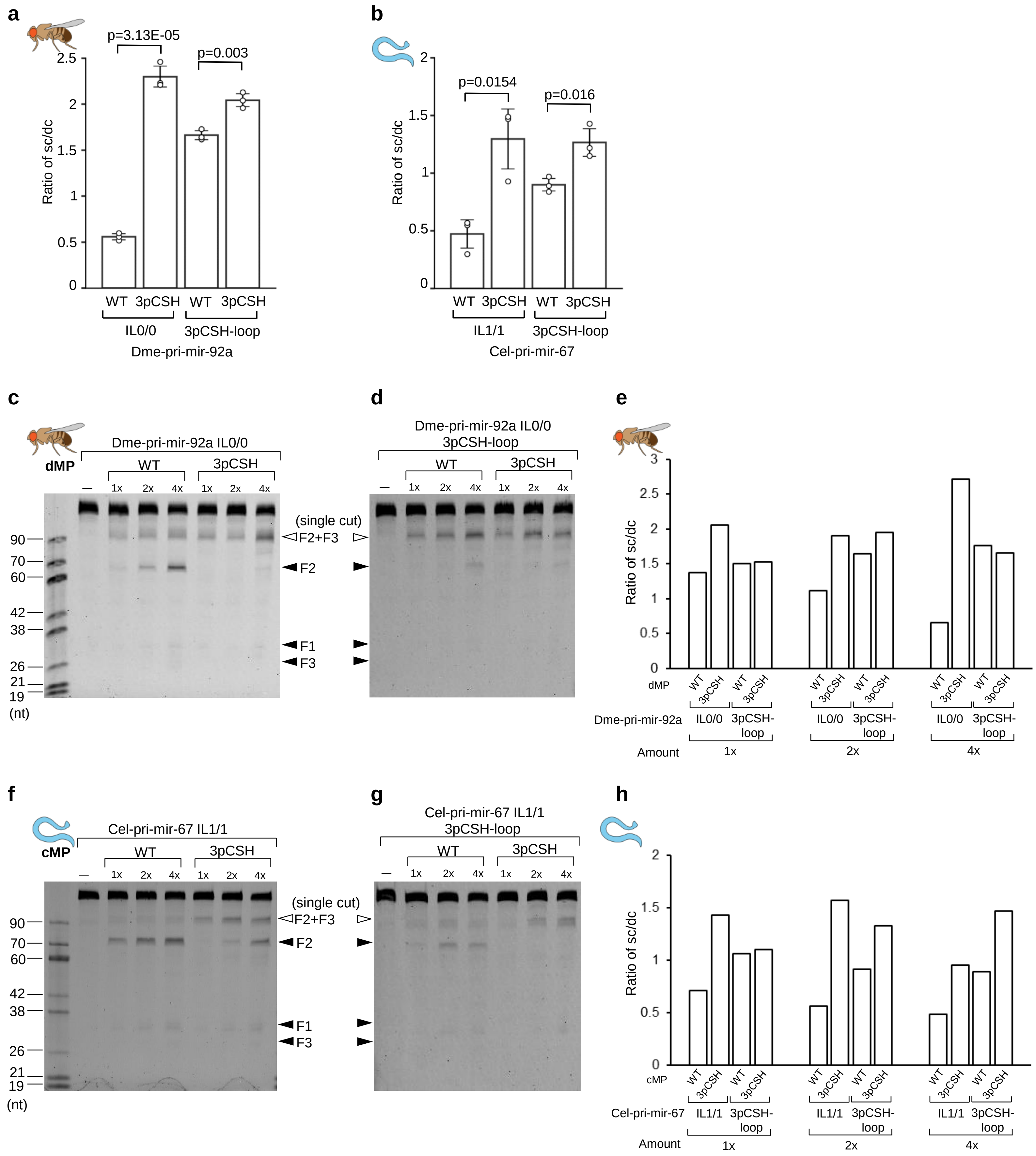

Supplementary Fig. 7 3pCSH is essential for the 3p-cleavage of animal DROSHAs. **a, b**) The sc/dc ratio was calculated as the ratio of the single-cut (F2+F3) to double-cut product (F2) band density for three repeated pri-miRNA cleavage results as shown in Fig. 6g, i. The p-values of the two-tailed t-test for the sc/dc ratios estimated from three replicates were shown. The error bars represent SEM. **c, d, f, g**) Pri-miRNA titration cleavage assays of dMP and cMP. The three different amounts of dMP- or cMP-bound IgG beads (0.75, 1.5, and 3 μL) were incubated with 3 pmol of pri-miRNAs. **e, h**) The sc/dc ratio was calculated as the ratio of the single-cut (F2+F3) to double-cut product (F2) for the pri-miRNA cleavage results as shown in (c), (d), (f), and g).

2b

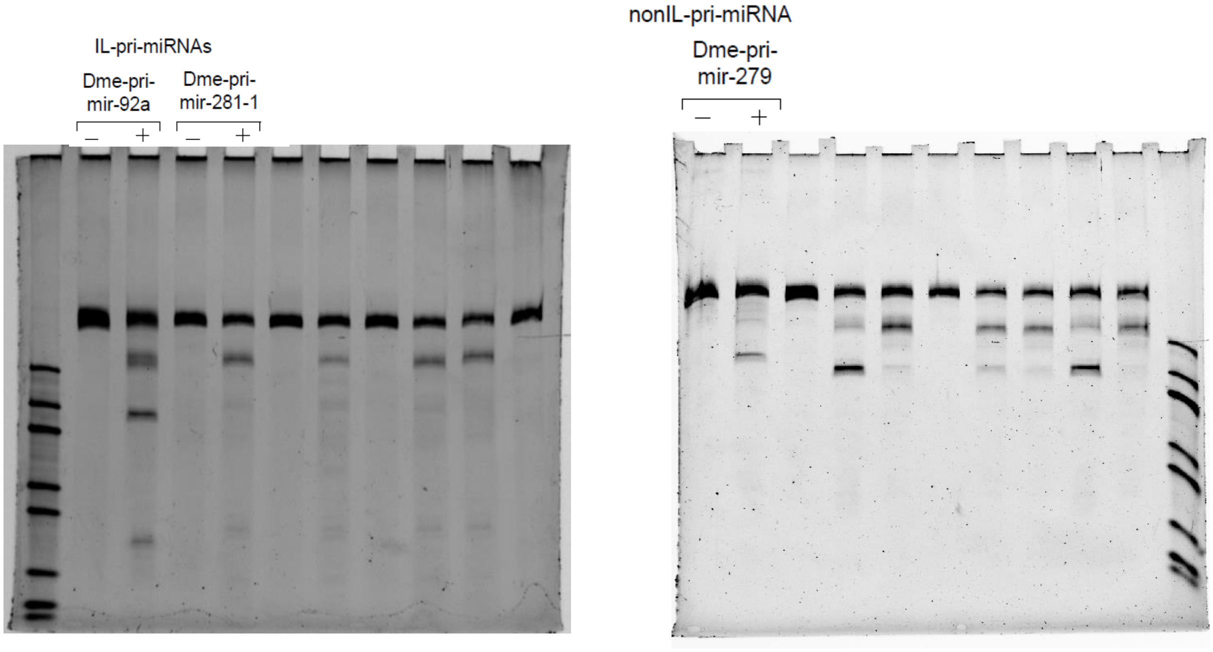

2d

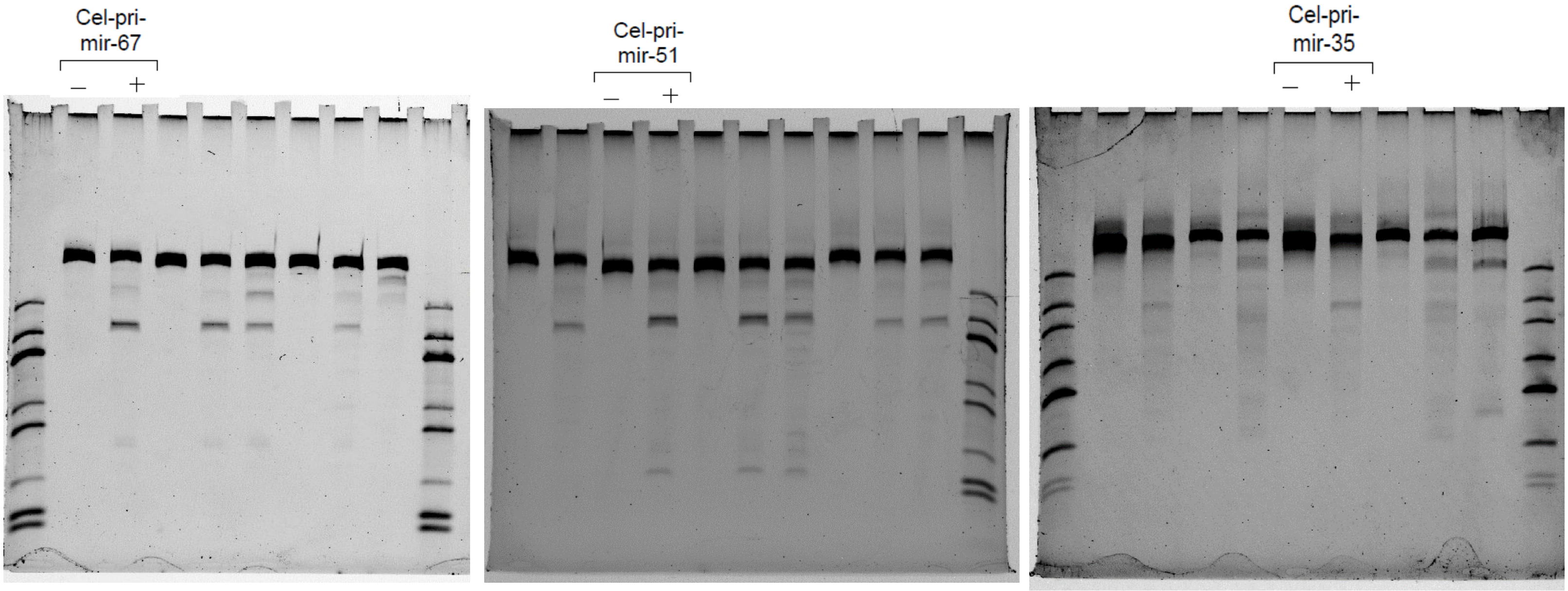

3b

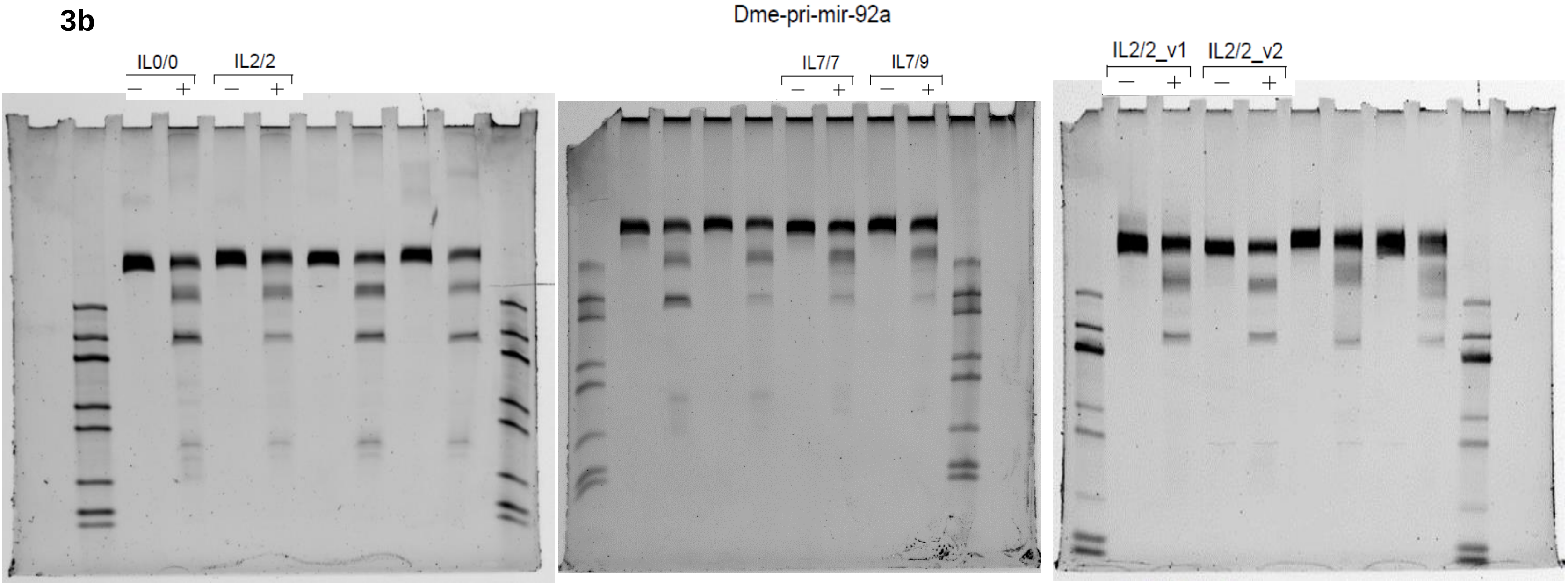

Supplementary Fig. 8 Uncropped gel images of Fig. 2b, 2d, and 3b.

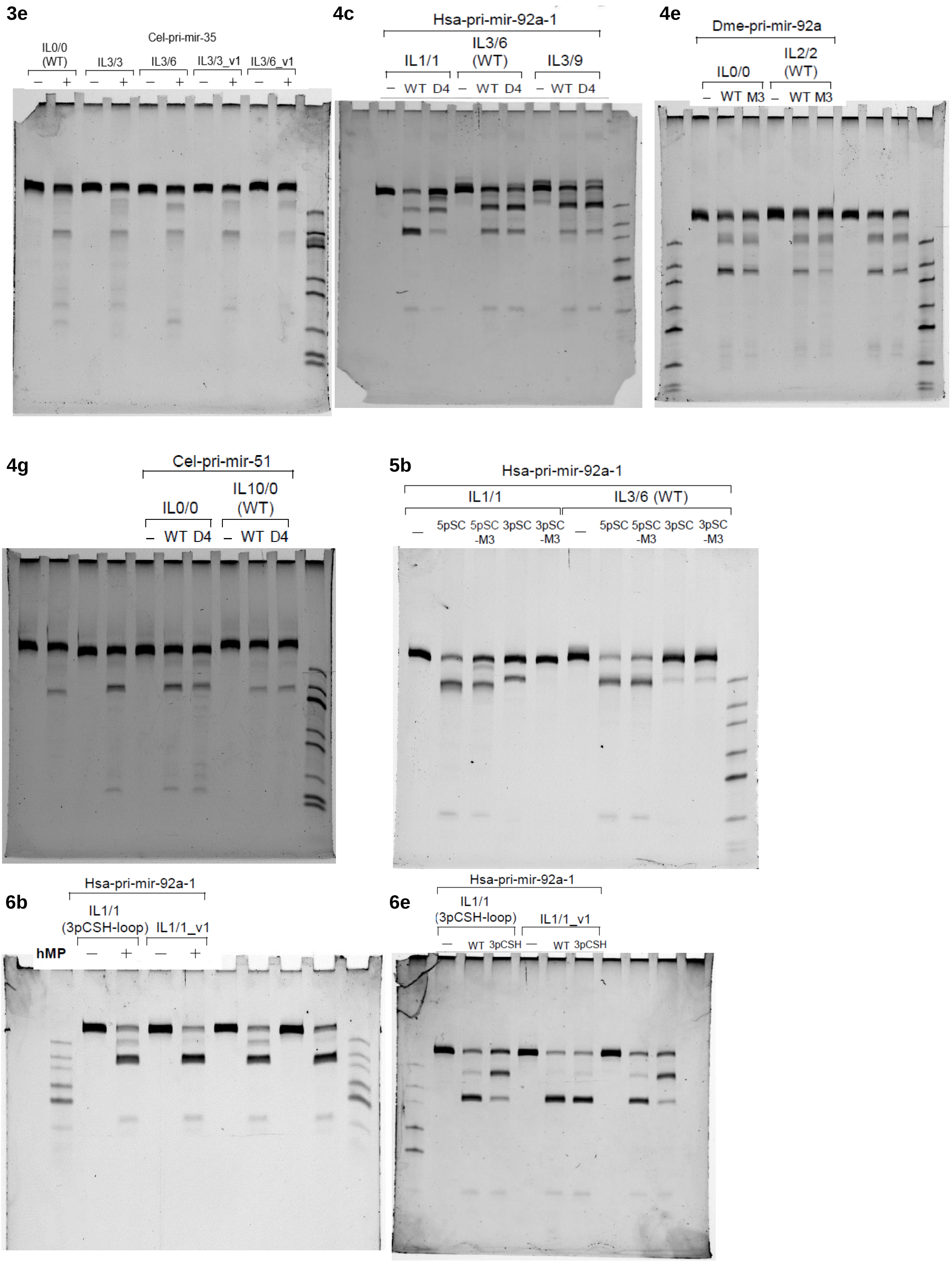

Supplementary Fig. 9 Uncropped gel images of Fig. 3e, 4c, 4e, 4g, 5b, 6b, and 6e.

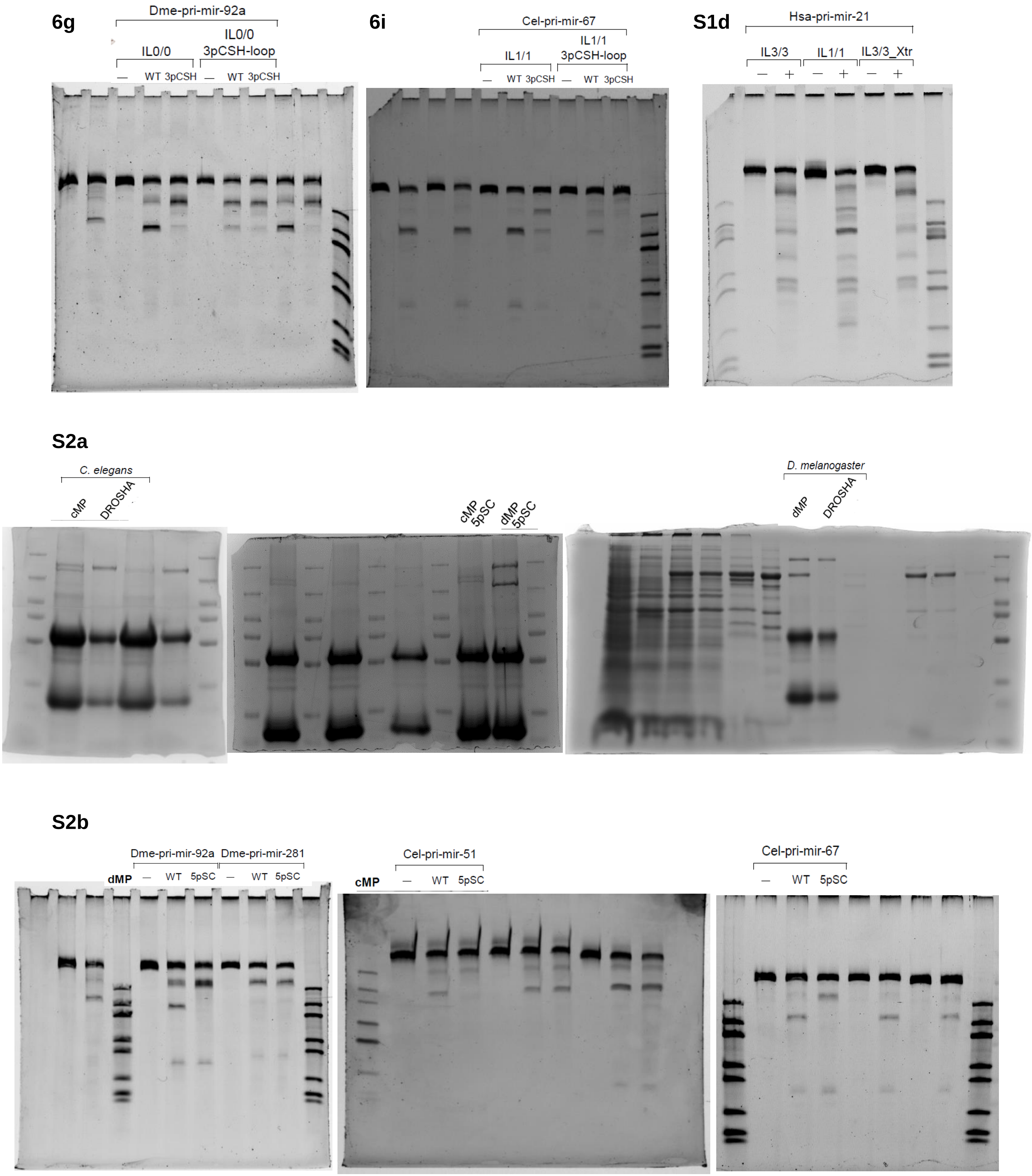

Supplementary Fig. 10 Uncropped gel images of Fig. 6g, 6i, Supplementary Fig 1d (S1d), Supplementary Fig. 2a (S2a), and Supplementary Fig. 2b (S2b).

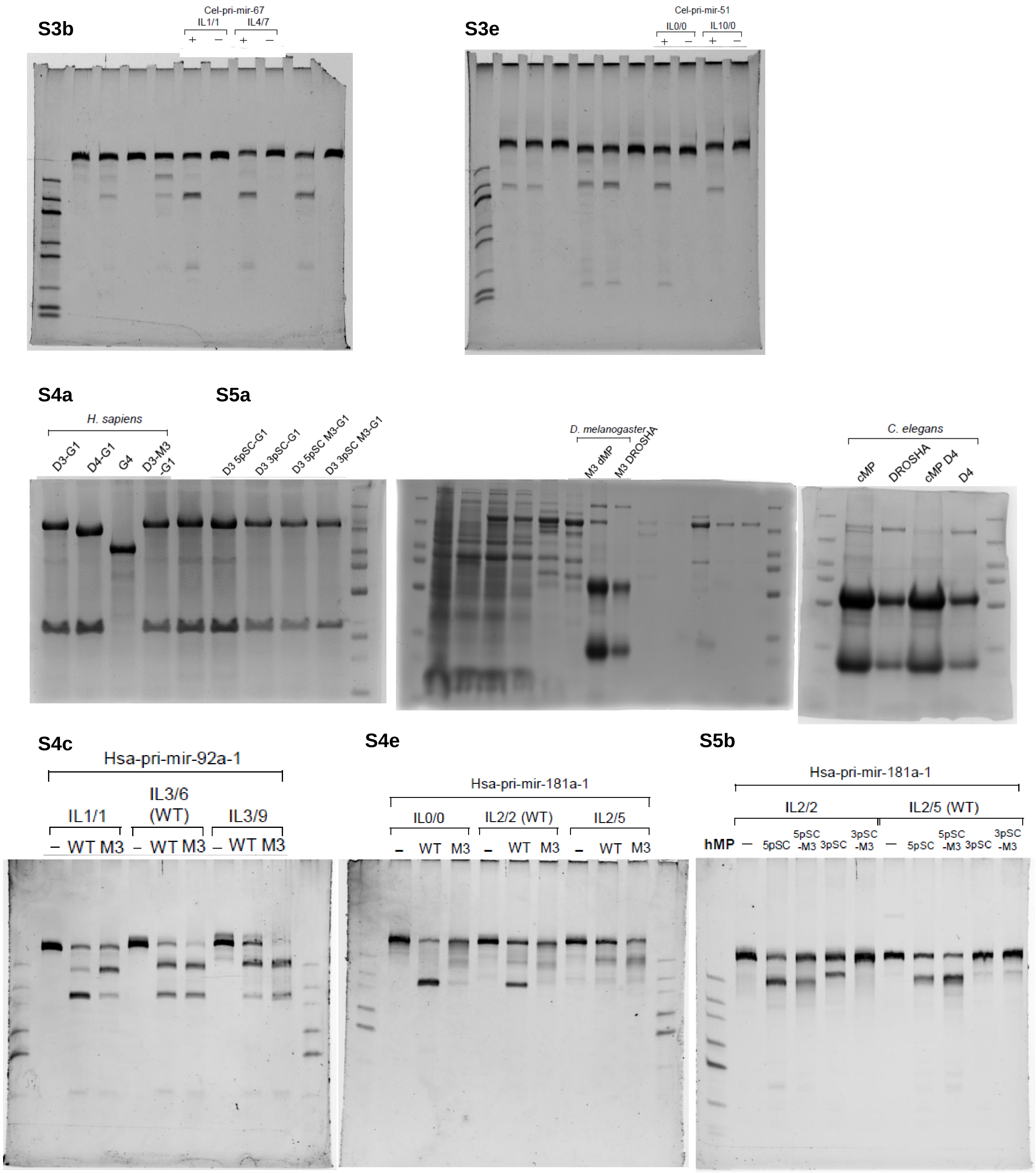

Supplementary Fig. 11 Uncropped gel images of Supplementary Fig. 3b (S3b), Supplementary Fig. 3e (S3e), Supplementary Fig. 4a (S4a), Supplementary Fig. 4c (S4c), Supplementary Fig. 4e (S4e), Supplementary Fig. 5a (S5a), and Supplementary Fig. 5b (S5b).

S5c

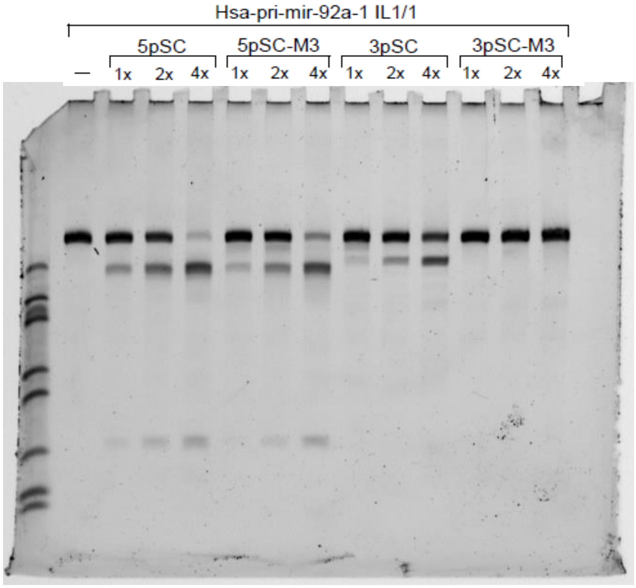

S5d

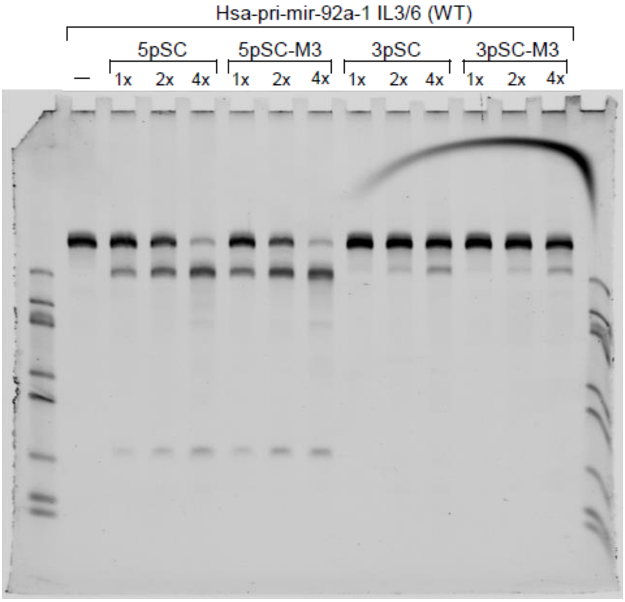

S6b

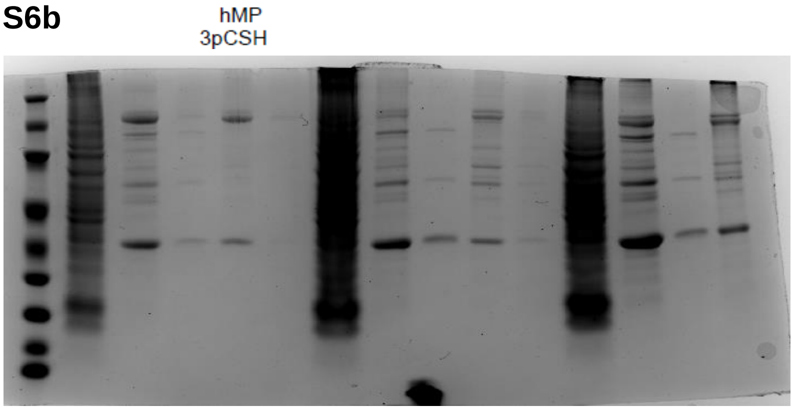

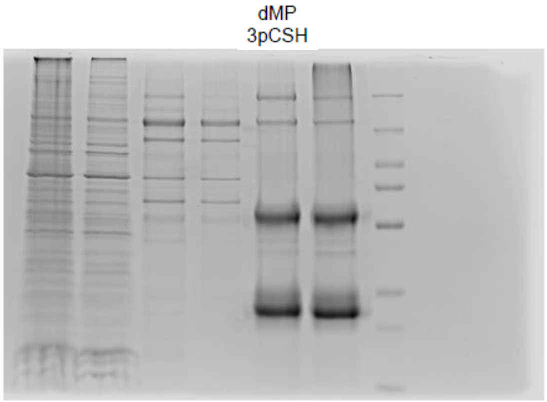

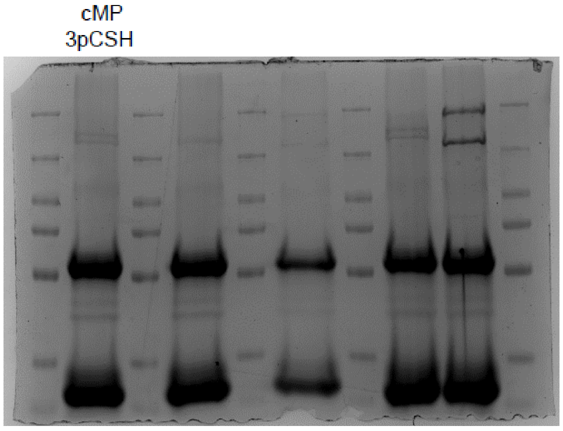

S6d

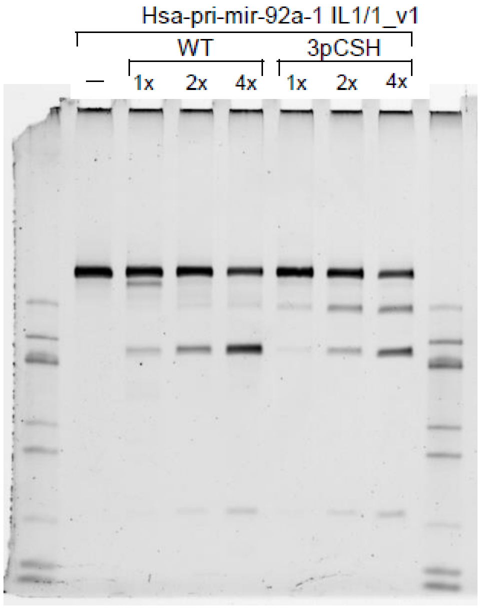

Supplementary Fig. 12 Uncropped gel images of Supplementary Fig. 5c (S5c), Supplementary Fig. 5d (S5d), Supplementary Fig. 6b (S6b), and Supplementary Fig. 6d (S6d).

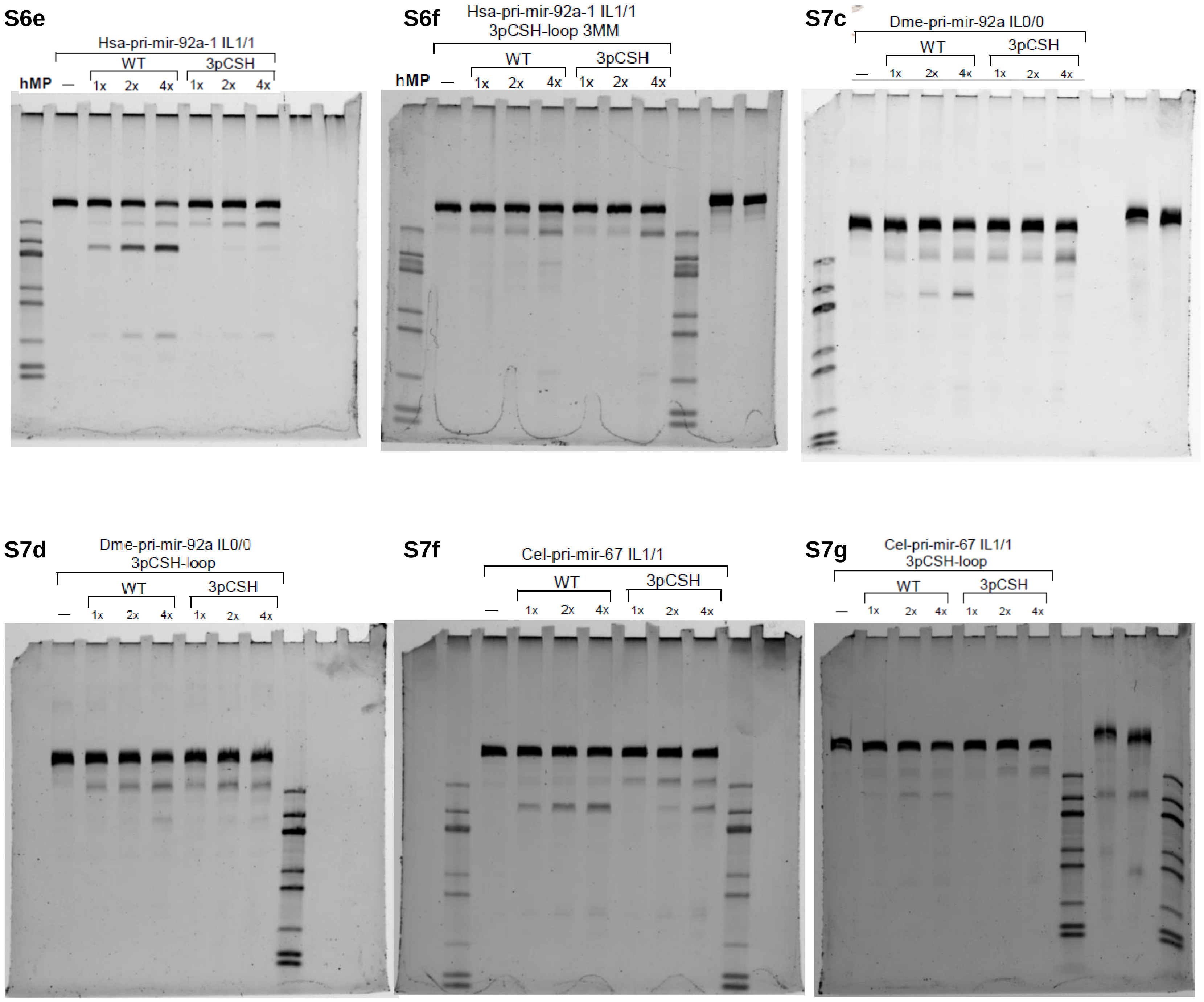

Supplementary Fig. 13 Uncropped gel images of Supplementary Fig. 6e (S6e), Supplementary Fig. 6f (S6f), Supplementary Fig. 7c (S7c), Supplementary Fig. 7d (S7d), Supplementary Fig. 7f (S7f), and Supplementary Fig. 7g (S7g).

Supplementary Table 1. The primer sequences used in cloning DROSHA and DGCR8

Cloning primer	Sequence (5'-3')	Purpose	Method of cloning	Plasmid
F-pXab-Cel-DROSHA	CGATCGGGATCCATGTCGGACGAAAAGATT	Amplify Cel-DROSHA	In-fusion cloning (Takara)	pXab-Cel-DROSHA
R-pXab-Cel-DROSHA	CAGTCTAGAGTCGCTGGTATCTTGTTC			
F-vector-Cel-DROSHA	AGCGACTCTAGACTGGAAGTTCTCTTCC	Amplify pXab backbone		
R-vector-Cel-DROSHA	CATGGATCCCGATCGGGTACCCTGGA			
F-pXG-Cel-PASH-1	CTCTCTAGAATGGAACAAGAATCTGGC	Amplify Cel-PASH-1	In-fusion cloning (Takara)	pXG-Cel-PASH-1
R- pXG-Cel-PASH-1	AAGTCTAGATTTGTGTGGTTTCTGATG			
F-vector-Cel-PASH-1	CACAAATCTAGACTTGAAGTTCTCTTTC	Amplify pXG backbone		
R-vector-Cel-PASH-1	TTCCATTCTAGAGAGCTCGAGGTCG			
F-Fly DROSHA-SalI	CGCGTCGACATGTACCAGCCGCCTTTG	Amplify Dme-DROSHA	SalI and XbaI digestion and T4 DNA ligation (NEB)	pXab-Dme-DROSHA
R-Fly DROSHA-XbaI	GGCTCTAGATCCCAGCGAAGATTTTG			
F-Fly DGCR8-SalI	CGCGTCGACATGGCGGAGAAGCCGCTG	Amplify Dme-PASHA	SalI and XbaI digestion and T4 DNA ligation (NEB)	pXG-Dme-PASHA
R-Fly DGCR8-XbaI	CCGTCTAGAAAGTTCCACGTTGTTCAAATTG			

Supplementary Table 2. Primer sequenses used in mutagenesis

Mutagenesis primer	Sequence (5'-3')	Template	Resulting plasmid
F-D3-M3	CACTTAGCACAGAAGGAAAAGGAGGTAGC AGTCCTCTGTACAAGACTCTGC	pXab-D3	pXab-D3-M3
R-D3-M3	CTTCTGTGCTAAGTGTCCTGGCACAGATCG CAAGCTGGGATTTGGGGTCATTCC		
F-D3-M3	CACTTAGCACAGAAGGAAAAGGAGGTAGC AGTCCTCTGTACAAGACTCTGC	pXab-D3-TN1 (Also pXab-D3-5pSC) (Nguyen et al., 2020)	pXab-D3-M3-5pSC
R-D3-M3	CTTCTGTGCTAAGTGTCCTGGCACAGATCG CAAGCTGGGATTTGGGGTCATTCC		
F-D3-M3	CACTTAGCACAGAAGGAAAAGGAGGTAGC AGTCCTCTGTACAAGACTCTGC	pXab-D3-TN2 (Also pXab-D3-3pSC) (Nguyen et al., 2020)	pXab-D3-M3-3pSC
R-D3-M3	CTTCTGTGCTAAGTGTCCTGGCACAGATCG CAAGCTGGGATTTGGGGTCATTCC		
F-pXab-D4	ATCAGGATTGGAATTCTAGACTGGAAGTTC TC	pXab-D3	pXab-D4
R-pXab-D4	AATTCCAATCCTGATTCAAAATGAACTCT		
F-Drosha-E1171A-G1172A	GCAGCACACTTAACTTTGTTGCGAAGCT	pXab-D3	pXab-D3-3pCSH
R-Drosha-E1171A-G1172A	AGTTAAGTGTGCTGCATGATGATCTGGGAA ATG		
F-Dme-DROSHA-5pSC	TTTCAAGCCCTAATGGGCGCGCTT	pXab-Dme-DROSHA	pXab-Dme-DROSHA-5pSC
R-Dme-DROSHA-5pSC	CATTAGGGCTTGAAAACAGTTGGCCATGGC		
F-Dme-DROSHA-M3	CTTAGTACAATGGATGGCGGCGGTGGATCT TCCCCCTACTACAAGGTG	pXab-Dme-DROSHA	pXab-Dme-DROSHA-M3
R-Dme-DROSHA-M3	ATCCATTGTACTAAGTGTACGAGCGCAAATT GCCAGCTTCGACTTGGG		
F-Dme-DROSHA-3pCSH	CACGCAGCACACTTGTCCCTGCTAC	pXab-Dme-DROSHA	pXab-Dme-DROSHA-3pCSH
R-Dme-DROSHA-3pCSH	CAAGTGTGCTGCGTGGTGCTCAGGGAAG		
F-Cel-DROSHA-5pSC	TTTCAAGCTGTGATGGCTGCAATA	pXab-Cel-DROSHA	pXab-Cel-DROSHA-5pSC
R-Cel-DROSHA-5pSC	CATCACAGCTTGAAAAGCATTAGCCAG		
F-Cel-DROSHA-dsRBDdel	AGCGAGAAATGGAATATGAGCTTTTCGAAG A	pXab-Cel-DROSHA	pXab-Cel-D4
R-Cel-DROSHA-dsRBDdel	ATTCCATTTCTCGCTTTCGATAAAGTGT		
F-Cel-DROSHA-3pCSH	GCAGCACATATGTCACTGCTACGAAC	pXab-Cel-DROSHA	pXab-Cel-DROSHA-3pCSH
R-Cel-DROSHA-3pCSH	TGACATATGTGCTGCGTGATGATATGGAAA GC		

Supplementary Table 3. Primer sequences used in pri-miRNA synthesis

Pri-miRNAs	Primer name	Primer sequence (5'-3')	Method
Hsa-pri-mir-21	F-T7-pri-21	TAATACGACTCACTATAGGGACATCTCCATGGCTGT	PCR from human genomic DNA
	R-pri-21	TCAGATGAAAGATACCAAAA	
Hsa-pri-mir-21 IL1/1	F-T7-pri-21	TAATACGACTCACTATAGGGACATCTCCATGGCTGT	PCR from hsa-pri-mir-21 DNA
	R-pri-21 IL1.1	TCAGATGAAAGATACCACCATGTCAGACAGCCCAT	
Hsa-pri-mir-21 IL3/3_Xtr	F-T7-pri-21 IL3.3_Xtr	TAATACGACTCACTATAGGGACATCTCCATGGCTGTACCATTCTGTCGGGTAGCTTAT	PCR from hsa-pri-mir-21 DNA
	R-pri-21	TCAGATGAAAGATACCAAAA	
Hsa-pri-mir-181a-1	F-T7-181a-1	TAATACGACTCACTATAGGGTCTGAGTTTTGAGGTTG	PCR from human genomic DNA
	R-181a-1	GTAGATGATGGTTAGCCATAG	
Hsa-pri-mir-181a-1 IL0/0	F-T7-181a-1	TAATACGACTCACTATAGGGTCTGAGTTTTGAGGTTG	PCR from hsa-pri-mir-181a-1 DNA
	R-181a-1 IL0/0	GTAGATGATGGTTAGCCTCAGGGTACAATCAACGG	
Hsa-pri-mir-181a-1 IL2/5	F-T7-181a-1	TAATACGACTCACTATAGGGTCTGAGTTTTGAGGTTG	PCR from hsa-pri-mir-181a-1 DNA
	R-181a-1 IL2/5	GTAGATGATGGTTAGCCATTTTAGGGTACAATCAACGG	
Hsa-pri-mir-92a-1	F-T7-mir-92a-1	TAATACGACTCACTATAGGGAAACTCAAACCCCTTTCTACAC	PCR from human genomic DNA
	R-92a1	TCACAATCCCCACCAAACTCA	
Hsa-pri-mir-92a-1 IL1/1	F-T7-mir-92a-1	TAATACGACTCACTATAGGGAAACTCAAACCCCTTTCTACAC	PCR from hsa-pri-mir-92a-1 DNA
	R-92a1-IL1/1	TCACAATCCCCACCCTCAACAGGCCGGGA	
Hsa-pri-mir-92a-1 IL3/9	F-T7-mir-92a-1	TAATACGACTCACTATAGGGAAACTCAAACCCCTTTCTACAC	PCR from hsa-pri-mir-92a-1 DNA
	R-92a-IL3/9	TCACAATCCCCACCAAAAAACTCAACAGGCCGG	
Dme-pri-mir-92a	F-T7-Dme-92a	TAATACGACTCACTATAGGGCCGAATATAAATATG	PCR from fly genomic DNA
	R-Dme-92a	GTTGTTTATTACAAACCG	
Dme-pri-mir-92a IL0/0	F-T7-Dme-92a	TAATACGACTCACTATAGGGCCGAATATAAATATG	PCR from Dme-pri-mir-92a DNA
	R-Dme-92a IL0/0	GTTGTTTATTACAAACTTCCCATAGGCCGGGAC	
Dme-pri-mir-92a IL7/7	FT7-Dme-92a IL7.7	TAATACGACTCACTATAGGGCCGAAtAtAAAcctGAAaaaaCCGtAGGACGGGAAG	PCR from Dme-pri-mir-92a DNA
	R-Dme-92a IL7/7	GTTGTTTATCCCATTTTTTCCATAGGCCGGGACA	
Dme-pri-mir-92a IL7/9	FT7-Dme-92a IL7.7	TAATACGACTCACTATAGGGCCGAAtAtAAAcctGAAaaaaCCGtAGGACGGGAAG	PCR from Dme-pri-mir-92a DNA
	R-Dme-92a IL7/9	GTTGTTTATCCCATTTTTTTTCCATAGGCCGGGACA	
Dme-pri-mir-92a IL0/0 3pCSH-loop	F-T7-Dme-92a	TAATACGACTCACTATAGGGCCGAATATAAATATG	PCR from Dme-pri-mir-92a DNA
	R-Dme-92a IL0/0 -1-2M	GTTGTTTATTACAAACTTCTTATAGGCCGGGACAAG	

Supplementary Table 3 (Continued).

Pri-miRNAs	Primer name	Primer sequence (5'-3')	Method
Dme-pri-mir-281-1	FT7-Dme-281-1	TAATACGACTCACTATAGGGCGAATCCAAATGCGA	PCR from fly genomic DNA
	R-Dme-281-1	CAGCGCATCGTCGAATAT	
Dme-pri-mir-279	FT7-Dme-279	TAATACGACTCACTATAGGGAATTGGAATTCA	PCR from fly genomic DNA
	R-Dme-279	TTGATGATTGAACTACCG	
Cel-pri-mir-35	F-T7-Cel-mir-35	TAATACGACTCACTATAGGGTTATTCTCGGATCAGATCGAGCCATTGCTGGTTTCTTCCACAGTGGTACTTTCCA	1 cycle extention
	R-Cel-mir-35	GCAAGTGGAAAAGATCGAGCCACTGCTAGTTTCCACCCGGTGATAGTTCTAATGGAAAGTACCACTGTGGAA	
Cel-pri-mir-35 IL3/3	F-T7	TAATACGACTCACTATAGGG	PCR from Cel-pri-mir-35 DNA
	R-Cel-mir-35-IL3/3	GCAAGTGGAAAAGATCTTTCCACTGCTAGTTTCCA	
Cel-pri-mir-35 IL3/6	F-T7	TAATACGACTCACTATAGGG	PCR from Cel-pri-mir-35 DNA
	R-Cel-mir-35-IL3/6	GCAAGTGGAAAAGATCTTTTTTCCACTGCTAGTTTCCA	
Cel-pri-mir-51	FT7-Cel-mir-51	TAATACGACTCACTATAGGGTGCTCACTTGTCCGAAAAGTCCGTCTACCCGTAGCTCCTATCCATGTTACTGGTC	1 cycle extention
	R-Cel-mir-51	GCTTCATGACCCTACTCGCCGTGCACCTGTACCTGCTTCCATGTTCACTTTTTGACCAGTAACATGGATAGGATA	
Cel-pri-mir-51 IL0/0	FT7-Cel-mir-51 IL0/0	TAATACGACTCACTATAGGGTGCTCACTTGCCGTCTACCCGTAGCTCC	1 cycle extention
	R-Cel-mir-51	GCTTCATGACCCTACTCGCCGTGCACCTGTACCTGCTTCCATGTTCACTTTTTGACCAGTAACATGGATAGGATA	
Cel-pri-mir-67	F-T7-Cel-mir-67	TAATACGACTCACTATAGGGCAACTCGATCAAAGA	PCR from worm genomic DNA
	R-Cel-mir-67	AATTCAAAGTTTTAAAATCGATCTACTCTTTCTAGG	
Cel-pri-mir-67 IL1/1	F-T7-Cel-Mir-67	TAATACGACTCACTATAGGGCAACTCGATCAAAGA	PCR from Cel-pri-mir-67 DNA
	R-Cel-mir-67 IL1/1	AATTCAAAGATTAATCGATCTACTCTTT	
Cel-pri-mir-67 IL1/1 3pCSH-loop	F-T7-Cel-Mir-67	TAATACGACTCACTATAGGGCAACTCGATCAAAGA	PCR from Cel-pri-mir-67 DNA
	R-Cel-mir-67 IL1/1 M-1-2	AATTCAAAGATTAATCTTTCTACTCTTTCTAGG	
