# Supplementary material for: The conserved single-cleavage mechanism of animal DROSHA enzymes
Source: Commun Biol. 2021 Nov 25;4:1332. doi: 10.1038/s42003-021-02860-1 (PMC8616936; doi:10.1038/s42003-021-02860-1)
Supplement: Supplementary file 2 — Description of Additional Supplementary Files [file 42003_2021_2860_MOESM2_ESM.pdf]

Description of Additional Supplementary Files

File name: Supplementary Data 1

Description: Information of 9630 pri-miRNAs.

File name: Supplementary Data 2

Description: The orthologous pri-miRNAs of human IL-pri-miRNAs.

File name: Supplementary Data 3

Description: Source data for Figures 1d–e, 3c, f, 4d, f, h, 5c–d; and Supplementary Figures 1e, 3c, f, 5e–f, 6c, g, 7a–b, e, h.
